# A critical re-evaluation of the slope factor of the operational model of agonism: When to exponentiate operational efficacy

**DOI:** 10.1038/s41598-023-45004-7

**Published:** 2023-10-16

**Authors:** Alena Randáková, Dominik Nelic, Jan Jakubík

**Affiliations:** Institute of Physiology Czech Academy of Sciences, Vídeňská 1083, 142 20 Praha, Prague, Czech Republic

**Keywords:** Drug discovery, Pharmacodynamics, Receptor pharmacology

## Abstract

Agonist efficacy denoting the “strength” of agonist action is a cornerstone in the proper assessment of agonist selectivity and signalling bias. The simulation models are very accurate but complex and hard to fit experimental data. The parsimonious operational model of agonism (OMA) has become successful in the determination of agonist efficacies and ranking them. In 1983, Black and Leff introduced the slope factor to the OMA to make it more flexible and allow for fitting steep as well as flat concentration–response curves. First, we performed a functional analysis to indicate the potential pitfalls of the OMA. Namely, exponentiation of operational efficacy may break relationships among the OMA parameters. The fitting of the Black & Leff equation to the theoretical curves of several models of functional responses and the experimental data confirmed the fickleness of the exponentiation of operational efficacy affecting estimates of operational efficacy as well as other OMA parameters. In contrast, fitting The OMA based on the Hill equation to the same data led to better estimates of model parameters. In conclusion, Hill equation-based OMA should be preferred over the Black & Leff equation when functional-response curves differ in the slope factor. Otherwise, the Black & Leff equation should be used with extreme caution acknowledging potential pitfalls.

## Introduction

In pharmacology, efficacy serves as a measure of how much response each ligand-receptor complex can produce upon its formation^1^. A sound method to assess agonist efficacy is essential for research and drug discovery. The operational model of agonism (OMA) introduces the term operational efficacy^2^. OMA has become a golden standard in the evaluation of agonism and subsequently also of signalling bias^3–5^. The OMA describes the response of the system as a function of ligand concentration using three parameters: (1) The equilibrium dissociation constant of agonist (K_A_) to the receptor initiating functional response; (2) The maximal possible response of the system (E_MAX_); (3) The operational efficacy of agonist (τ). As we will show, in the OMA, K_A_ represents the affinity of the agonist for the receptor. Therefore, K_A_ is specific to a given combination of ligand and receptor. The maximal possible response of the system E_MAX_ is specific to the system. The operational efficacy (τ) is a measure of the response to a given agonist at a given system, ranging from 0 to infinity, and is specific to a combination of ligand and system.

OMA has numerous limitations and possible pitfalls. All three parameters (E_MAX_, K_A_, and τ) are inter-dependent, thus, one of them has to be predetermined before fitting OMA to data or the global fit of multiple curves at various receptor densities has to be performed^6^. Besides that, there are additional methodological and conceptual issues^7–9^. Here we focus on the exponentiation of operational efficacy. In practice, positive cooperativity or positive feedback leads to steep and negative cooperativity or negative feedback leads to flat concentration–response curves that the OMA (Eq. (2)) does not fit. Therefore, an equation intended for the description of non-hyperbolic concentration curves (Eq. (7)) was introduced by Black et al.^10^. Since then, Eq. (7) has been commonly used. The presented simple mathematical analysis of Eq. (7) shows that the slope factor affects the relationship between observed maximal response to agonist (E’_MAX_) and operational efficacy (τ) and the relationship between the concentration of agonist for half-maximal response (EC_50_) and its equilibrium dissociation constant (K_A_). Some combinations of operational efficacy and slope factor in the Black & Leff equation (Eq. (7)) lead to EC_50_ greater than K_A_ which is biochemically counterintuitive. In the system with receptor reserve maximum effect is reached before the occupation of all receptors is reached. Therefore, half of the effect (EC_50_) is reached before reaching the occupation of half of all receptors (K_A_). In a system without receptor reserve, full occupation of receptors is reached before reaching system E_MAX_. Therefore, EC_50_ occurs at K_A_. Black & Leff model, however, allows for EC_50_ > K_A_ in the systems with low efficacy and high slope factor.

The Hill equation was originally formulated to describe the binding of oxygen molecules to haemoglobin^11,12^. The Hill equation was then incorporated into the first model of receptor function, the so-called occupation theory^13^. In contrast to the Black et al. slope factor, the slope factor of the Hill equation (Eq. (10)) does not affect the centre or asymptotes of the hyperbola describing concentration–response curves. Therefore, the Hill coefficient does not affect relationships between E’_MAX_ and τ nor between EC_50_ and K_A_. This makes Hill equation-based OMA (Eq. (11)) more practical in many ways. Fitting the Black & Leff equation (Eq. (7)) to the theoretical data revealed several drawbacks, like under- or over-estimation of parameters or high levels of uncertainty of parameter estimates. Moreover, in contrast to Hill equation-based OMA (Eq. (11)), in some cases, fitting the Black & Leff equation to the experimental data resulted in the wrong ranking of agonist efficacies τ and wrong estimates of agonist K_A_. However, it should be noted that the Black & Leff equation should not be used firsthandily as slopes of individual response curves vary among agonists.

### The general concept of the operational model of agonism

For demonstrative purposes, we will derive OMA from scratch for receptor-effector systems. This will become handy for the analysis of systems with low expression of receptors. The OMA equation for ligand-gated ion channels is the same, although, it is based on different sets of equations. In general, OMA consists of two functions. One function describes the binding of an agonist to a receptor as the dependence of the concentration of agonist-receptor complexes [RA] on the concentration of an agonist [A]. The second function describes the dependence of functional response (E) on the concentration of agonist-receptor complexes [RA]. OMA expresses the dependence of response E on the concentration of [A].

### Rectangular hyperbolic OMA

#### Definition of OMA

In the simplest case, when both binding and response functions are described by rectangular hyperbola (Supplementary Information Figure S1), the resulting function is also a rectangular hyperbola. For example, in a bi-molecular reaction, the dependence of ligand binding to the receptor [RA] is described by Eq. (1) where [A] is the concentration of ligand and K_A_ is its equilibrium dissociation constant that represents the concentration of ligand at which half of the total number of receptors, R_T_, binds the ligand and the other half of the receptors is free.

1 $$\left[ {RA} \right] = \frac{{R_{T} \left[ A \right]}}{{\left[ A \right] + K_{A} }}$$

If the bound ligand is an agonist, it activates the receptor and produces functional response E. Response as a function of agonist binding (agonist-receptor complexes [RA]) is given by Eq. (2).

2 $$E = \frac{{E_{MAX} \left[ {RA} \right]}}{{\left[ {RA} \right] + K_{E} }}$$

where E_MAX_ is the maximum possible response of the system and K_E_ is the value of [RA] that elicits a half-maximal effect. Various agonists produce a functional response of different strengths. The OMA was postulated to introduce the “transducer ratio” τ that is given by Eq. (3).

3 $$\tau = \frac{{R_{T} }}{{K_{E} }}$$

The substitution of Eq. (2) with Eq. (1) and Eq. (3) gives Eq. (4).

4 $$E = E_{MAX} \frac{\left[ A \right]\tau }{{\left[ A \right]\left( {\tau + 1} \right) + K_{A} }}$$

#### Analysis of OMA

Equation (4) is the equation of OMA^2^. It has three parameters: The equilibrium dissociation constant of agonist (K_A_) at the effect-eliciting (active) state of the receptor^6^ that is specific to a combination of ligand and receptor. The maximal possible response of the system (E_MAX_) is specific to the system. And the “transducer ratio” (τ) that is specific to a combination of ligand and system. Equation (4) is a rectangular hyperbola with the horizontal asymptote, the observed maximal response to agonist A (E’_MAX_), given by Eq. (5).

5 $$E_{MAX}^{\prime } = E_{MAX} \frac{\tau }{\tau + 1}$$

A more efficacious agonist (having a high value of parameter τ) elicits higher E’_MAX_ than less efficacious agonists (having a low value of parameter τ). Thus, τ is actually operational efficacy. The relationship between parameter τ and E’_MAX_ is hyperbolic meaning that two highly efficacious agonists (e.g., τ values 10 and 20) differ in E’_MAX_ values less than two weak agonists (e.g., τ values 0.1 and 0.2).

In Eq. (4), the concentration of agonist A for half-maximal response (EC_50_), is given by Eq. (6).

6 $${\text{EC}}_{50} = { }\frac{{{\text{K}}_{{\text{A}}} }}{{{\uptau } + 1}}$$

According to Eq. (6), for τ > 0, the EC_50_ value is always lower than the K_A_ value. The K_A_ to EC_50_ ratio is greater for efficacious agonists than for weak agonists. Similarly to E’_MAX_, the relationship between parameter τ and EC_50_ is hyperbolic. In contrast to E’_MAX_ values, the ratio K_A_ to EC_50_ ratio is more profound for two highly efficacious agonists (e.g., τ values 10 and 20) than for two weak agonists (e.g., τ values 0.1 and 0.2).

#### Limitations of OMA

The OMA has several weak points. The major drawback of OMA is the lack of physical basis of the agonist equilibrium dissociation constant K_A_. Equation (2) assumes that agonist binding [RA] denotes effect-producing active complexes. The agonist binding to the receptor in an inactive conformation is not observed in the response (K_E_ → ∞; τ = 0). In the radioligand binding experiments, agonists bind to all receptor conformations including the inactive ones. For various reasons, the receptors in an active conformation may be scarce or absent from radioligand binding experiments. Then it may be impossible to determine the K_A_ value in the radioligand binding experiments. All three parameters of OMA (E_MAX_, K_A_ and τ) are interdependent^6^. To fit Eq. (4) to individual concentration–response curves, one of the parameters must be fixed. E.g., the maximal response of the system E_MAX_ is determined by comparing the functional response to a given agonist in a system with a reduced population of receptors by irreversible alkylation^14^ or cell lines with varying receptor expression levels^6^. Alternatively, global fitting of multiple concentration–response curves with all parameters free can be employed. However, due to a high number of degrees of freedom global fitting is less robust than *per partes* methods^6^. Another limitation of the OMA is that the shape of the functional response is a rectangular hyperbola.

### Non-hyperbolic OMA

#### Definition of non-hyperbolic OMA

In practice, concentration–response curves steeper or flatter than the ones described by Eq. (4) are observed. In such cases, Eq. (4) does not fit experimental data. As stated by the authors, Eq. (7) was devised for non-hyperbolic dependence of functional response on the concentration of agonist^10^. Eq. (7) was derived in the same way as Eq. (4) from Eq. (1) and Eq. (2) while [RA] and K_E_ in Eq. (2) were exponentiated to factor **n**.

7 $${\text{E}} = { }\frac{{\left[ {\text{A}} \right]^{{\text{n}}} {\uptau }^{{\text{n}}} {\text{E}}_{{{\text{MAX}}}} }}{{\left[ {\text{A}} \right]^{{\text{n}}} {\uptau }^{{\text{n}}} + { }\left( {\left[ {\text{A}} \right] + {\text{ K}}_{{\text{A}}} } \right)^{{\text{n}}} }}$$

#### Analysis of non-hyperbolic OMA

Introduced power factor **n** changes the slope and shape of the functional-response curve (Supplementary information Figure S3). Nevertheless, Eq. (7) as a mathematical function has rectangular asymptotes: The horizontal asymptote (*x* →  ± ∞) E’_MAX_ is given by Eq. (8) and the vertical asymptote (*y* →  ± ∞) is equal to -K_A_/(τ + 1) (Supplementary Information Eq. (S2) and Eq. (S3)). From Eq. (7), the EC_50_ value is given by Eq. (9).

8 $$E^{\prime}_{MAX} = E_{MAX} \frac{{\tau^{n} }}{{1 + \tau^{n} }}$$

9 $${\text{EC}}_{50} = {\text{K}}_{{\text{A}}} { }\frac{1}{{\sqrt[{\text{n}}]{{2 + {\uptau }^{{\text{n}}} }}{ } - { }1}}$$

**Figure 1:**
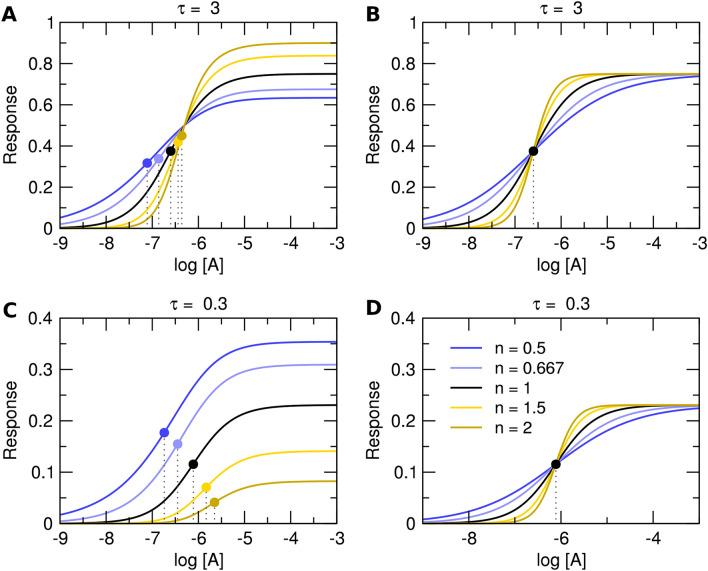
Theoretical concentration–response curves. Theoretical curves of concentration responses under equilibrium according to Eq. (7), left and Eq. (11), right. Simulation parameters: E_MAX_ = 1; τ = 3 (top) or τ = 0.3 (bottom); K_A_ = 10^−6^ M. Values of slope factors are listed in the legend.

**Figure 2:**
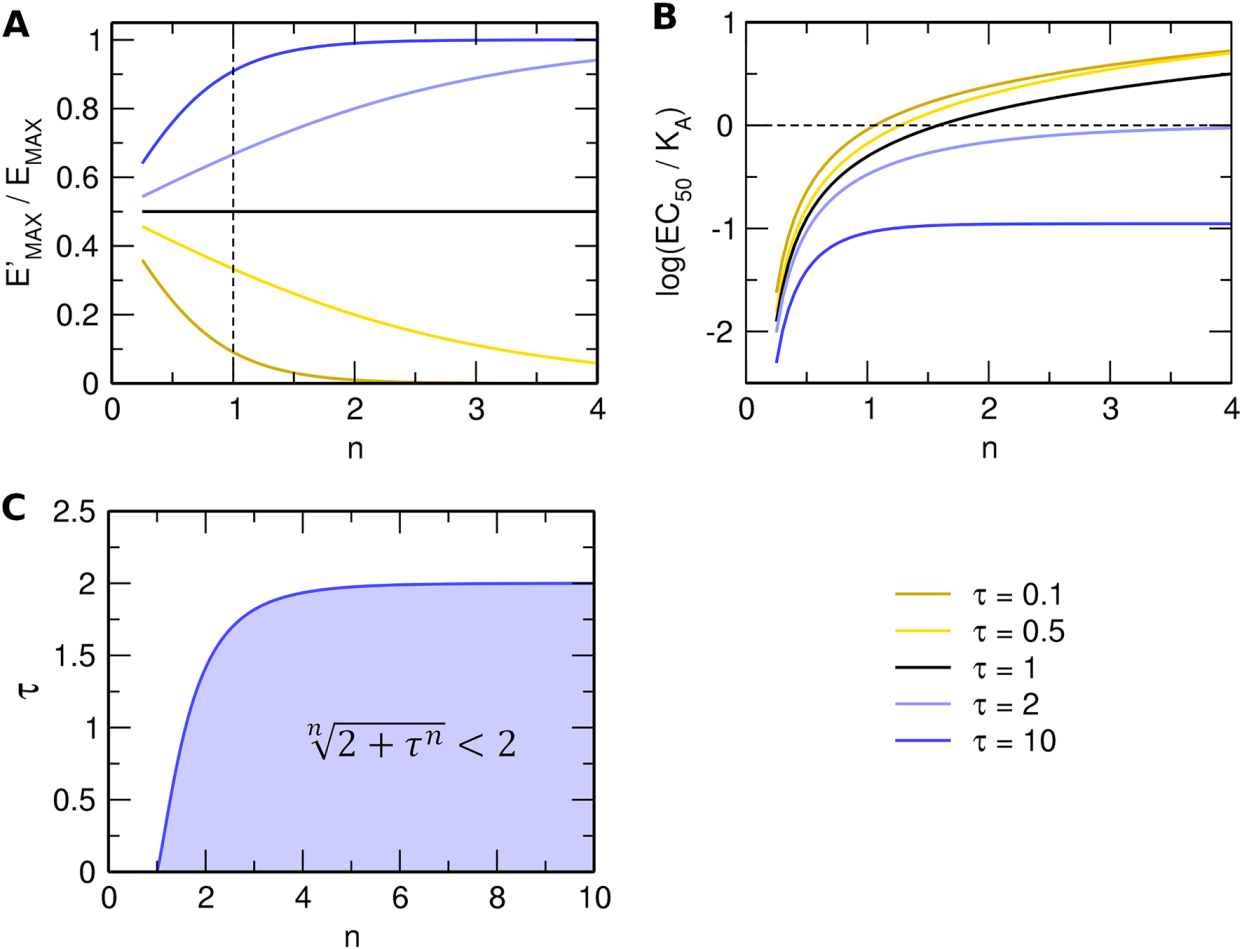
Analysis of Black & Leff equation (Eq. (7)). (**A**) Dependency of observed E’_MAX_ to system E_MAX_ ratio (ordinate) on slope factor **n** (abscissa) and operational efficacy τ (legend). (**B**) Dependency of EC_50_ to K_A_ ratio (ordinate) on slope factor **n** (abscissa) and operational efficacy τ (legend). (**C**) Inequality plot of slope factor **n** (abscissa) and operational efficacy τ (ordinate) yielding half-efficient concentration EC_50_ greater than equilibrium dissociation constant K_A_.

Evidently, the introduced slope factor **n** affects both the observed maximal response E’_MAX_ and the half-efficient concentration of agonist EC_50_ ( Fig. 1A and C). The influence of the slope factor on E’_MAX_ is **bidirectional** (Supplementary Information Table S1, Figure S2). For operational efficacies τ > 1, an increase in the value of the slope factor increases E’_MAX_. (Figs. 1A and 2A blue lines). For operational efficacies τ < 1, an increase in slope factor decreases E’_MAX_ (Figs. 1C and 2A yellow lines). The effect of the slope factor on E’_MAX_ is the most apparent for low values of operational efficacy τ, making the estimation of model parameters of weak partial agonists impractical. Imagine strong agonist τ = 10 and weak agonist τ = 0.1. For n = 1: Strong agonist E’_MAX_ is 90% and weak agonist E’_MAX_ is 10% of system E_MAX_. For n = 2: Strong agonist E’_MAX_ is 99% (one-tenth more) and weak agonist E’_MAX_ is just 1% (ten times less).

An increase in the value of the slope factor increases the EC_50_ value (Fig. 2B). Again, the effect of the slope factor on the EC_50_ value is more eminent at low values of operational efficacy τ (yellow lines). Paradoxically, any combination of operational efficacy τ and slope factor fulfilling the inequality in Fig. 2C (blue area) results in EC_50_ values **greater** than K_A_ (e.g., Fig. 1C, yellow lines). For example, EC_50_ > K_A_ applies if τ = 0.5 and n > 1.6, or if τ = 1 and n > 1.6, or when τ = 1.5 and n > 2.15. The upper asymptote of inequality is 2. Thus, the possibility of EC_50_ > K_A_ applies to τ < 2 in these cases, due to the high slope factor **n** the response function (Eq. (2)) has a “lag” before the steep growth where the binding function (Eq. (1)) grows faster. Therefore, in this range EC_50_ > K_A_.

The operational efficacy τ may be also considered as a measure of “receptor reserve”. In a system with a relatively small capacity of a functional response output, the strong agonist reaches its maximal response before reaching full receptor occupancy. Thus, the agonist EC_50_ value is lower than its affinity for the receptor. The smaller the occupancy fraction needed for the full response to a given agonist the greater is difference between agonist EC_50_ and K_A_ values. According to OMA (Eq. (2)), the relation between EC_50_ and K_A_ is described by Eq. (6). The greater value of operational efficacy τ, the smaller EC_50_ value and the greater the difference from K_A_. Thus, the value of operational efficacy τ is a measure of the receptor reserve of a given agonist in a given system. In a system with a large capacity of functional output, agonists do not have a receptor reserve and must reach full receptor occupancy to elicit a full signal. In such a system, even strong agonists produce small effects attributed to partial agonists. Thus, the parameter τ is specific to a combination of ligand and system.

Nevertheless, for agonists that elicit at least some response in a given system, the parameter τ must be greater than 0. Then according to Eq. (6) of the operational model of agonism, the EC_50_ value must be smaller than the K_A_ value. In principle, the EC_50_ value greater than the K_A_ can be achieved only by some parallel mechanism that increases the apparent K’_A_ provided that a ratio of K’_A_ to K_A_ is greater than EC_50_ to K_A_. For example, such a mechanism may be negative allosteric modulation of agonist binding or non-competitive inhibition of functional response.

#### Limitations of the non-hyperbolic OMA

Besides all limitations of the hyperbolic OMA, the non-hyperbolic version of OMA has additional drawbacks. The most important is the lack of mechanistic background for factor **n**. Exponentiation of agonist concentration [A] to power factor **n** results in non-hyperbolic functional-response curves. Importantly, as shown above, exponentiation of operational efficacy τ to power factor **n** breaks the logical relationship between observed maximal response E’_MAX_ and operational efficacy τ. That, as it will be shown later, impedes the correct estimation of τ. Further, exponentiation of τ may result in K_A_ values smaller than EC_50_ (Fig. 2C, blue area).

### OMA with Hill coefficient

#### Definition of OMA with Hill coefficient

The Hill coefficient may serve as an alternative slope factor in the OMA. Hill equation incorporates the Hill coefficient as a slope factor to rectangular hyperbola^12^. The major advantage of the Hill coefficient as a slope factor is that it allows for a change in the eccentricity (vertices) of the hyperbola-like curves without changing the centre (EC_50_) and asymptotes (E’_MAX_) (Supplementary Information Figure S3). The Hill equation published in 1910^11^ can be formulated as^15^ Eq. (10).

10 $$E = \frac{{\left[ A \right]^{{n_{H} }} E_{MAX}^{\prime } }}{{\left[ A \right]^{{n_{H} }} + EC_{50}^{{n_{H} }} }}$$

where n_H_ is the Hill coefficient. Substitution of E’_MAX_ by Eq. (5) and EC_50_ by Eq. (6) gives Eq. (11). For derivation from first principles see for example Roche et al.^16^

11 $$E = \frac{{\left[ A \right]^{{n_{H} }} \frac{\tau }{\tau + 1}E_{MAX} }}{{\left[ A \right]^{{n_{H} }} + \left( {\frac{{K_{A} }}{\tau + 1}} \right)^{{n_{H} }} }}$$

As expected, the Hill coefficient does not influence the maximal observed response E’_MAX_ or half-efficient concentration of agonist EC_50_ (Fig. 1B,D). Eq. (11) was suggested as suitable for the analysis of functional responses displaying symmetrical response curves^16^.

#### Implications of OMA with Hill coefficient

Analysis of the OMA with slope factor by Black et al. (Eq. (7)) has shown that the slope factor **n** has a bidirectional effect on the relationship between the parameters E’_MAX_ and τ. and that the slope factor **n** affects the relationship between the parameters EC_50_ and K_A_. In contrast, in Eq. (11) neither the value of E’_MAX_ nor EC_50_ is affected by the Hill coefficient (Fig. 1B,D). The parameters E’_MAX_ and EC’_50_ can be readily obtained by fitting Eq. (10) to the single concentration–response data.

#### Limitations of OMA with Hill coefficient

The major criticism of the Hill equation is its parsimonious character. It is relatively simple and its parameters are easy to estimate. However, as a model, it is just an approximation. In an experiment, the slope of the concentration–response curve different from unity may be a result of the parallel signalling mechanism providing feedback or allosteric cooperativity. In the case of positive cooperativity, it results in steep concentration–response curves. In the case of negative cooperativity, it results in flat concentration–response curves.

### OMA of allosteric systems

The simplest scenario leading to variation in the slope of concentration–response curves is allosteric interaction between agonist and allosteric modulator or two molecules of agonist, e.g., in a ligand-gated channel or dimeric GPCR^17,18^. Positive cooperativity among agonist molecules results in steep functional-response curves and negative cooperativity results in flat functional-response curves. As the mode of cooperativity is the property of a given agonist, the slope of the functional-response curve may vary among agonists. Slope factor **n** in Eq. (7) is deemed the property of the system and thus the same for all agonists. In the attempt to keep slope factor **n** constant in allosteric systems, the second slope factor **m** was introduced to Eq. (7) resulting in^19^ Eq. (12):

12 $$E = \frac{{\left[ A \right]^{nm} \tau^{n} E_{MAX} }}{{\left[ A \right]^{nm} \tau^{n} + \left( {\left[ A \right]^{m} + K_{A}^{m} } \right)^{n} }}$$

where factor **n** links agonist concentration to the slope of the functional-response curve and factor **m** links agonist concentration to the slope of the receptor-binding curve. As the slope factors are interdependent one of them must be predetermined. It is only possible to predetermine the slope of the binding curve **m**. When fitting Eq. (12) to the data, functional-response slope factor **n** should become the same for all agonists. Observed maximal response E’_MAX_ is still given by Eq. (8) but half-efficient concentration EC_50_ is given by Eq. (13).

13 $$EC_{50} = K_{A} \sqrt[m]{{\frac{1}{{\sqrt[n]{{2 + \tau^{n} }} - 1}}}}$$

Similarly to Eq. (9), the effect of the binding slope factor **m** on the EC_50_ to K_A_ ratio depends on operational efficacy τ and functional response slope factor **n**. The effect of binding slope factor **m** depends on the radicand value. The m-th root is greater than the radicand when **m** > 1 and radicand > 1 or **m** < 1 and radicand < 1. The m-th root is smaller than the radicand when **m** < 1 and radicand > 1 or **m** > 1 and radicand < 1. Moreover, functional responses of allosteric systems may be not only steep or flat but also biphasic including bell-shaped^18^. Neither Eq. (7) nor Eq. (12) can accommodate such shapes and equations adequate to the mode of action are needed.

### OMA of non-competitive inhibition

**Figure 3:**
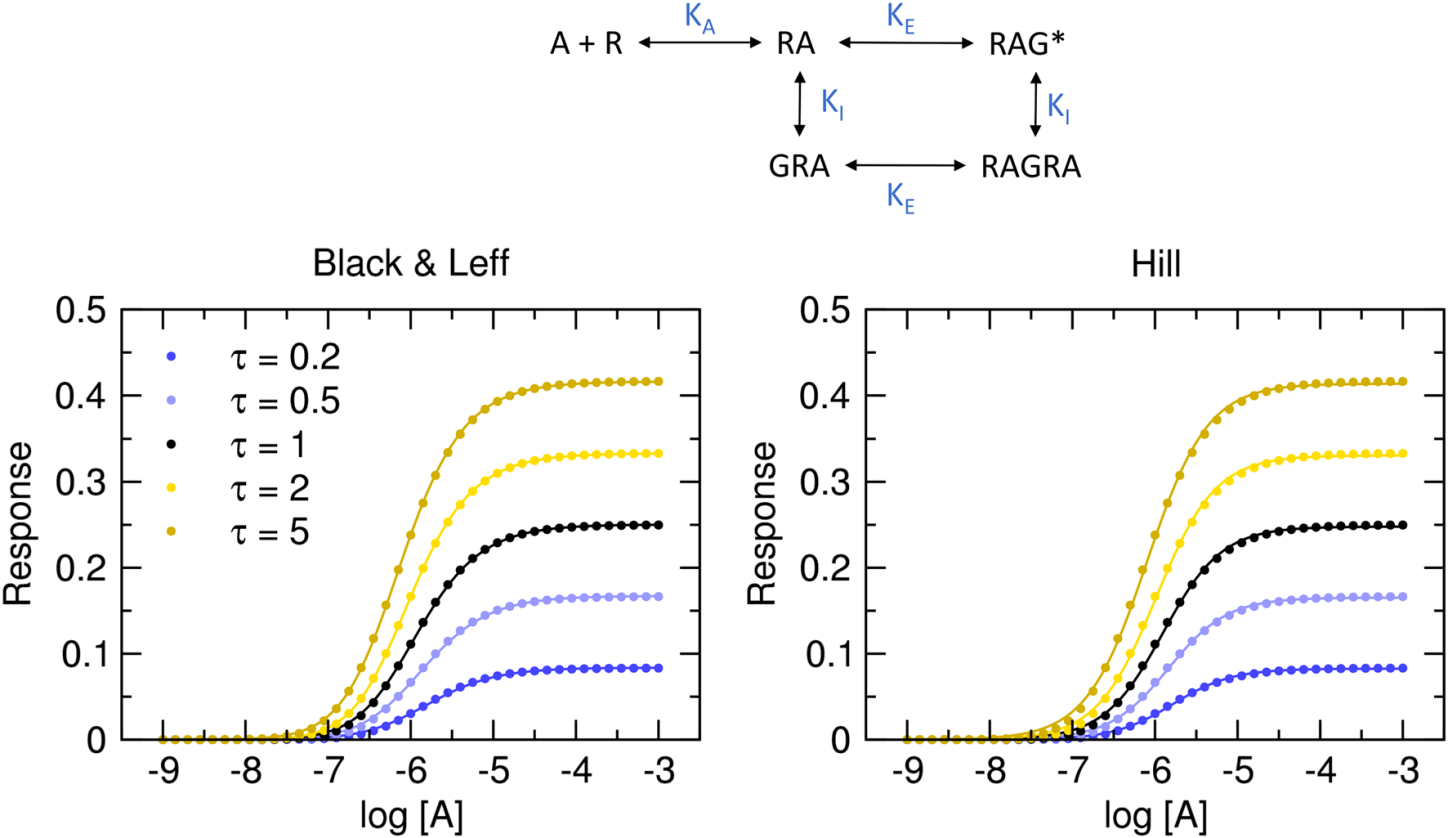
Non-competitive auto-inhibition of functional response. Dots, functional response to an agonist (K_I_ = 1, E_MAX_ = 1, R_T_ = 1, K_A_ = 10^−6^ M) in the model of non-competitive autoinhibition according to Eq. (15). Values of operational efficacies τ are indicated in the legend. Full lines, left, Black & Leff equation (Eq. (7)), right, Hill equation (Eq. (10)) fitted to the data. Parameter estimates are in Table 1.

As shown in Fig. 2, OMA with slope factor **n** allows for EC_50_ values higher than K_A_. The simplest mode of interaction that increases observed EC_50_ above K_A_ is non-competitive auto-inhibition^20–23^. Under non-competitive auto-inhibition, RA non-competitively blocks functional response by binding to effector G (Fig. 3). This type of inhibition is characterized by RA binding to a spatially distinct (allosteric) site resulting in a decreased response of effector G. In non-competitive inhibition, RA binds to both sites independently, exerting neutral binding cooperativity (absence of allosteric interaction). Non-competitive auto-inhibition results in a concentration-dependent increase in EC_50_ and a decrease in E’_MAX_. Functional response is given by Eq. (14).

14 $$E = E_{MAX} \frac{{\left[ {RA} \right]}}{{K_{E} + \left[ {RA} \right]}} \frac{{\left[ {RA} \right]}}{{K_{I} + \left[ {RA} \right]}}$$

For K_I_ > 0, after the substitution of Eq. (1) for [RA], Eq. (14) becomes Eq. (15) (Supplementary information Eq. (S31)).

15 $$E = E_{MAX} \frac{\tau \left[ A \right]}{{K_{A} + \left[ A \right]\left( {1 + \tau } \right)}} \frac{\sigma \left[ A \right]}{{K_{A} + \left[ A \right]\left( {1 + \sigma } \right)}}$$

where σ = R_T_/K_I_. The apparent maximal response E’_MAX_ is given by Eq. (16) (Supplementary Information Eq. (S32)).

16 $$E^{\prime}_{MAX} = E_{MAX} \frac{\tau }{1 + \tau } \frac{\sigma }{1 + \sigma }$$

Thus, apparent operational efficacy τ’ is given by Eq. (17).

17 $$\tau^{\prime } = \frac{\sigma \tau }{{\sigma + \tau + 1}}$$

The EC_50_ value for the model of non-competitive auto-inhibition is given by Eq. (18) (Supplementary information Eq. (S36)).

18 $$EC_{50} = K_{A} \frac{{\sqrt {\sigma^{2} + 6\sigma \tau + 8\sigma + \tau^{2} + 8\tau + 8} + \sigma + \tau + 2}}{{2\left( {1 + \sigma } \right)\left( {1 + \tau } \right)}}$$

Non-competitive auto-inhibition decreases apparent maximal response E’_MAX_, increases observed half-efficient concentration EC_50_ and results in steep curves (Fig. 3). The resulting concentration–response curve is asymmetric with a typical slope factor of about 1.2 regardless of values of K_I_ and K_E_. Both models fit well. Fitting Hill equation gives correct estimates of apparent maximal response E’_MAX_ and thus correct estimates of apparent operational efficacy τ’ (Table 1). In contrast, for K_I_ ≥ 1, fitting the Black & Leff equation results in underestimated values of τ and pK_A_. Values of τ^n^ well approximate τ’ values.

**Table 1:** Results of fitting Black & Leff equations to the model of non-competitive auto-inhibition. Black & Leff (Eq. (7)) and Hill (Eq. (10)) equations were fitted to model data Eq. (15) with E_MAX_ fixed to 1 or E’_MAX_ confined <  = 1, respectively.

Model Eqs. 15 and 17					Black & Leff				Hill					Equation 18
τ	pK_A_	K_I_	τ’	n	τ	τ^n^	pK_A_	E’_MAX_	n_H_	pEC_50_	E’_MAX_	τ	pK_A_	pK_A_
0.2	6.00	1	0.091	1.33	0.166	0.0917	5.91	0.084	1.21	5.78	0.083	0.090	5.74	5.99
0.5	6.00	1	0.200	1.46	0.333	0.201	5.98	0.167	1.22	5.84	0.165	0.199	5.76	5.99
1	6.00	1	0.333	1.58	0.499	0.333	6.06	0.250	1.22	5.91	0.248	0.330	5.79	5.99
2	6.00	1	0.500	1.71	0.666	0.499	6.15	0.333	1.21	5.99	0.331	0.494	5.82	6.00
5	6.00	1	0.714	1.55	0.804	0.713	6.15	0.416	1.18	6.10	0.414	0.706	5.87	6.00

Fitting Eq. (15) with fixed system E_MAX_ to the model of functional response of non-competitive inhibition yields correct parameter estimates that are associated with the low level of uncertainty only when correct initial estimates of τ and σ are given (Supplementary Information Figure S7 and S8). In the case of K_I_ = 5 (Supplementary Information Figure S9), estimates of operational efficacy τ and inhibition factor σ are swapped pointing to the symmetry of Eq. (15). This symmetry makes calculation of τ and σ impossible as any τ and σ combination resulting in an appropriate apparent efficacy τ’ (Eq. (17)) corresponds well to a given functional-response data (Supplementary Information Figure S8). Fitting the Black & Leff equation (Eq. (7)) yields wrong estimates of K_A_ and underestimated values of τ. Importantly, the extent of underestimation varies. The τ of 0.2 was underestimated by 17%. The τ of 5 was underestimated sixfold. In contrast, the calculation of apparent operational efficacies from the fitting of the Hill equation (Eq. (10)) is very close.

### Signalling feedback

Signalling feedback occurs when outputs of a system are routed back as inputs^24^. Negative feedback is a very common auto-regulatory (auto-inhibitory) mechanism in nature, for example at G-protein coupled receptors^25^. Positive feedback also occurs in biology to propagate signals that would be otherwise dampened by other mechanisms, e.g. neuronal action potential. In the receptor-effector systems, an increase in output signal [RAG] proportionally either decreases (negative feedback) or increases (positive feedback) input [RA]^26^. Functional response in employing feedback mechanisms is then given by Eq. (19). For derivation see Supplementary Information Eqs. (S25) to (S37).

19 $$E = E_{MAX} \frac{{\tau \left[ A \right]\left( {\tau \left[ A \right] + \left[ A \right] + K_{A} } \right)}}{{\left[ A \right]^{2} \left( {\delta \tau + \tau^{2} + \tau + 1} \right) + \left[ A \right]K_{A} \left( {\delta \tau + \tau + 2} \right) + K_{A}^{2} }}$$

where δ is a feedback factor. Values greater than 1 denote negative feedback. Values smaller than 1 denote positive feedback. The maximal response E’_MAX_ to an agonist with operational efficacy τ is related to the maximal response of the system E_MAX_ according to Eq. (20).

20 $$E{^\prime}_{MAX} = E_{MAX} \frac{{\tau^{2} + \tau }}{{\delta \tau + \tau^{2} + \tau + 1}}$$

Negative feedback decreases the observed maximal response to an agonist E’_MAX_ while positive feedback increases it. And apparent operational efficacy τ’ of a given agonist can be calculated according to Eq. (21).

21 $$\tau^{\prime } = \frac{{\tau^{2} + \tau }}{\delta \tau + 1}$$

Furthermore, negative feedback decreases the K_A_ to EC_50_ ratio while positive feedback increases it (Supplementary Information Eq. (S54)). For derivations see Supplementary Information Eq. (S37) through Eq. (S54). In general, negative feedback results in flat functional-response curves as with the increase in signal output proportionally more of an agonist is needed for the same increase of the signal. Conversely, positive feedback results in steep functional-response curves as signal output proportionally increases signal input (Supplementary Information Figure S9).

**Figure 4:**
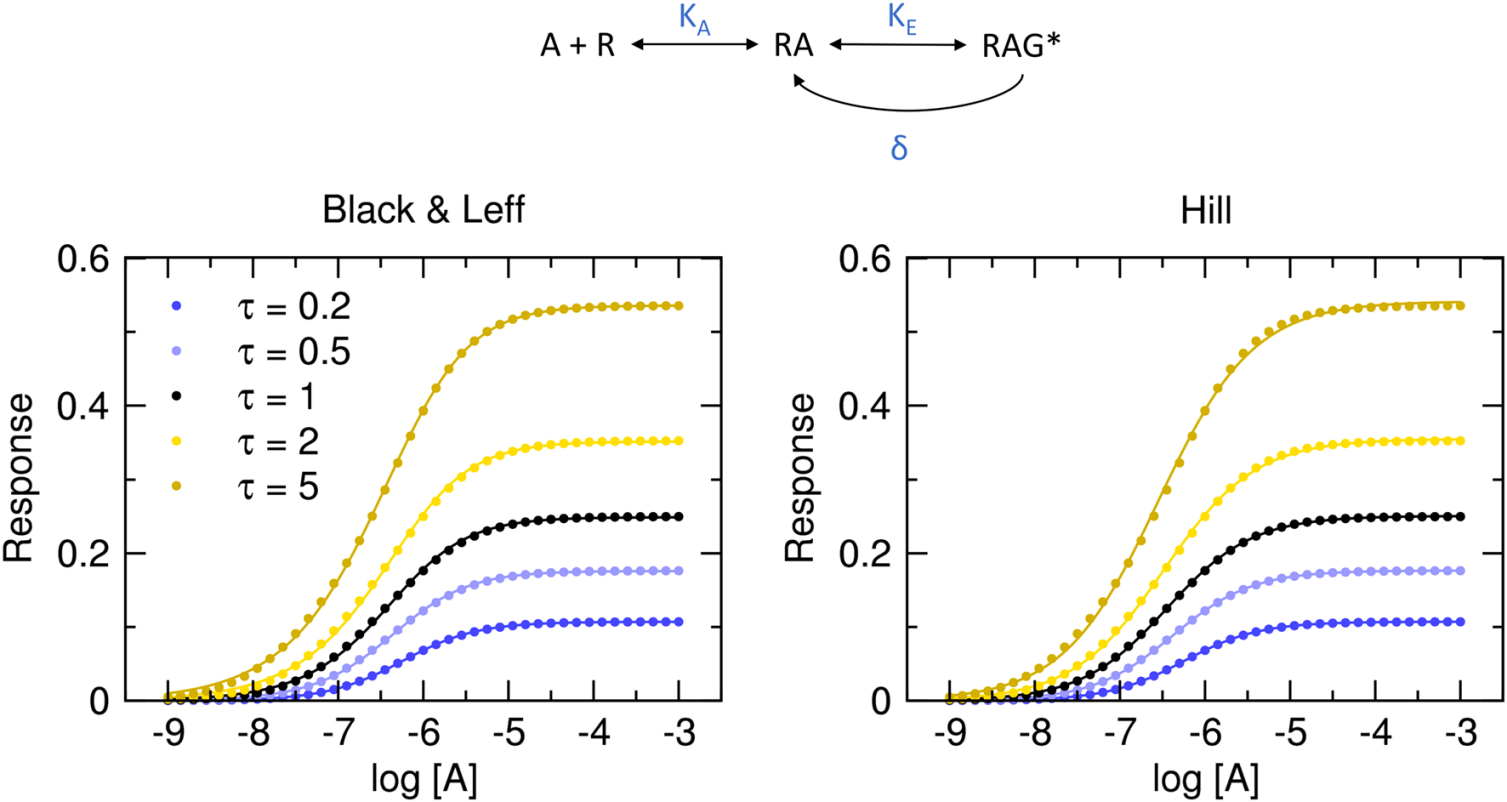
Functional response with signal feedback. Dots, functional response to an agonist (E_MAX_ = 1, R_T_ = 1, K_A_ = 10^−6^ M) in the model of the system with constant negative feedback (δ = 5) according to Eq. (19). Values of operational efficacies τ are indicated in the legend. Full lines, left, Black & Leff equation (Eq. (7)), right, Hill equation (Eq. (10)) fitted to the data. Parameter estimates are in Table 2.

Importantly, in a system with constant negative feedback, like in Fig. 4, the steepness of the curve depends on operational efficacy τ. Functional-response curves to agonists with high operational efficacy are flatter than the ones of agonists with low operational efficacy (Table 2).

**Table 2:** Results of fitting Black & Leff equations to the model of the system employing signalling feedback. Black & Leff (Eq. (7)) and Hill (Eq. (10)) equations were fitted to model data with E_MAX_ fixed to 1 or E’_MAX_ confined <  = 1, respectively.

Model Eqs. 19 and 21					Black & Leff				Hill					Eq. S42
τ	pK_A_	δ	τ’	n	τ	τ^n^	pK_A_	E’_MAX_	n_H_	pEC_50_	E’_MAX_	τ	pK_A_	pK_A_
0.2	6.00	5	0.120	0.96	0.109	0.119	6.17	0.106	0.98	6.25	0.107	0.120	6.20	6.01
0.5	6.00	5	0.214	0.87	0.168	0.212	6.20	0.175	0.93	6.38	0.177	0.214	6.30	6.01
1	6.00	5	0.333	0.76	0.236	0.334	6.13	0.250	0.87	6.45	0.251	0.333	6.33	6.02
2	6.00	5	0.545	0.68	0.409	0.544	6.01	0.353	0.81	6.48	0.355	0.545	6.29	6.01
5	6.00	5	1.154	0.66	1.245	1.16	5.86	0.536	0.78	6.55	0.541	1.155	6.22	6.01

Fitting Eq. (19) with fixed system E_MAX_ to the model employing feedback mechanisms yields correct parameter estimates that are associated with the low level of uncertainty when correct initial estimates of τ and δ are given (Supplementary Information Figure S9 and S10). Fitting the Black & Leff equation (Eq. (7)) yields wrong estimates of K_A_ and underestimated values of τ. Again, the extent of underestimation varies. The τ of 0.2 was underestimated by 67%. The τ of 5 was underestimated fourfold. However, values of τ^n^ well approximate τ’ values. In contrast, calculations of apparent operational efficacies from the fitting of the Hill equation (Eq. (10)) are very close.

### Systems with a similar expression of receptor and effector ([R_T_]≈[G_T_])

**Figure 5:**
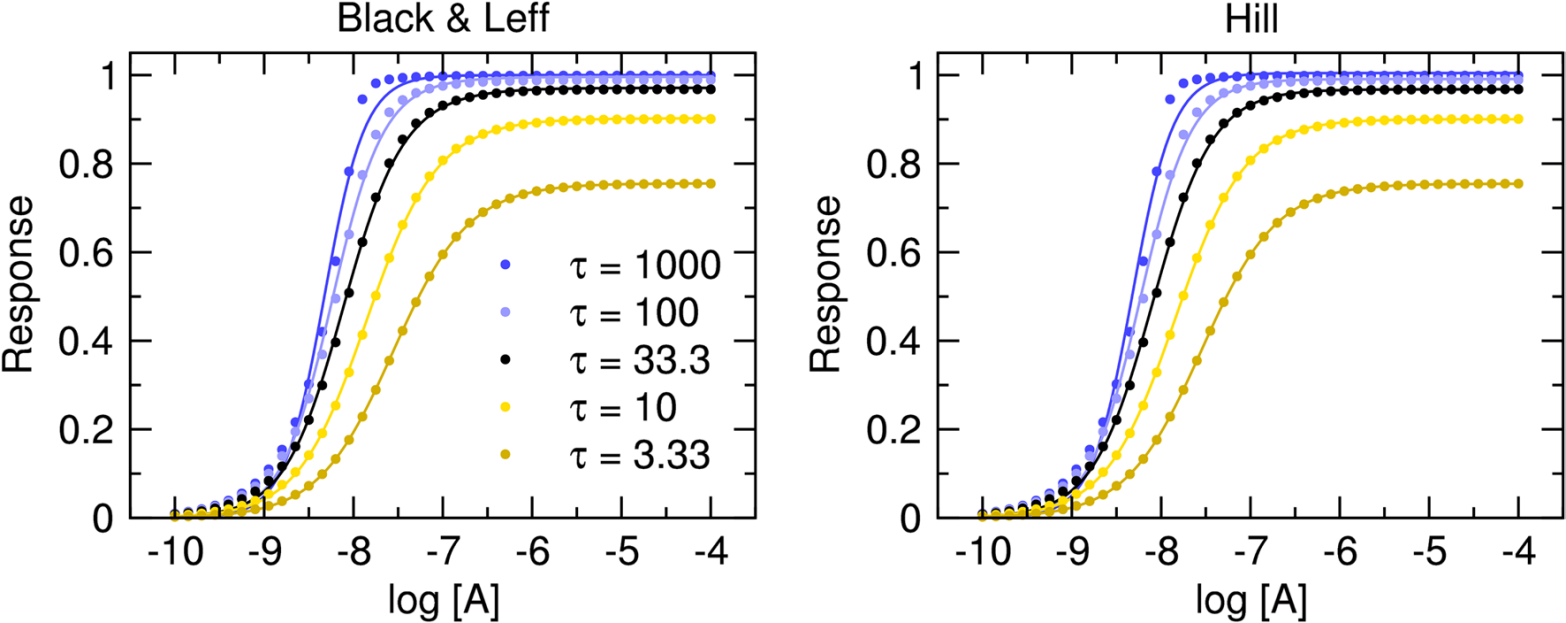
Functional response of the system with a similar expression of receptor and effector ([R_T_]≈[G_T_]). Dots, functional-response data modelled in two steps. First, binding was calculated according to Eq. (1). Then resulting [RA] was used in Eq. (22). E_T_ = 10^−6^ M, R_T_ = 10^−5^ M, K_A_ = 10^−7^ M. Values of operational efficacies τ are indicated in the legend. Full lines, left, Black & Leff equation (Eq. (7)), right, Hill equation (Eq. (10)) fitted to the data. Parameter estimates are in Table 3.

The Eq. (2) and consequently Eq. (4) are valid only when either [RA] or [G] is constant. That requires [R_T_] >  > [G_T_] or [R_T_] <  < [G_T_]. Usually, systems exhibit receptor reserve, indicating [R_T_] >  > [G_T_]. In systems with low receptor expression, [R_T_] and [G_T_] may be similar. In a such system [RAG] as a function of [RA] is given by Eq. (22) (Supplementary Information Eq. (S62)).

22 $$\left[ {RAG} \right] = \frac{1}{2} \left( {K_{E} + \left[ {RA} \right] + \left[ {G_{T} } \right] - \sqrt {K_{E}^{2} + 2K_{E} \left( {\left[ {RA} \right] + \left[ {G_{T} } \right]} \right) + (\left[ {RA} \right] - \left[ {G_{T} } \right])^{2} } } \right)$$

where [RA] denotes the concentration of all receptor agonist complexes, free RA plus RA in complex with G (RAG) (Supplementary Information Eq. (S56)). [RAG] as a function of [A] (Supplementary Information Eq. (S64)) has only approximate solutions. Therefore, [RA] values as a function of [A] were calculated as [RA] according to Eq. (1) and used in Eq. (22) to model functional responses of the system with low receptor-expression level (Fig. 5). The resulting curves are steep and asymmetric. In the low receptor-expression system, the response reaches the maximum at lower agonist concentration due to receptor depletion that cuts off the signal early which results in an apparent increased steepness and, in turn, in a curve asymmetry. The greater the operational efficacy is, the steeper and more asymmetric functional-response curves are (Table 3). However, the observed operational efficacy τ’ is equal to the modelled operational efficacy.

**Table 3:** Results of fitting Black & Leff equations to the model of the system with a similar expression of receptor and effector ([R_T_]≈[G_T_]). Black & Leff (Eq. (7)) and Hill (Eq. (10)) equations were fitted to model data with E_MAX_ fixed to 1 or E’_MAX_ confined <  = 1, respectively.

Model Eqs. 1 and 22			Black & Leff				Hill				
τ	pK_A_	n	τ	τ^n^	pK_A_	E’_MAX_	n_H_	pEC_50_	E’_MAX_	τ	pK_A_
1000	7.00	1.91	6701	2.03.10^7^	4.50	1.00	1.89	8.32	1.00	CNBD	CNBD
100	7.00	1.54	36.2	251	6.68	0.996	1.52	8.23	0.991	108	6.19
33.3	7.00	1.35	13.6	33.9	6.98	0.971	1.29	8.10	0.968	30.1	6.61
10	7.00	1.15	6.94	9.28	6.98	0.903	1.11	7.84	0.900	9.04	6.84
3.33	7.00	1.05	2.93	3.09	6.98	0.756	1.03	7.55	0.755	3.08	6.94

*CNBD* cannot be determined.

Fitting the Black & Leff equation (Eq. (7)) to the model system with a low receptor-expression level yields wrong estimates of K_A_ for extremely high operational efficacy (τ = 1000). Values of τ are far off for all modelled efficacies. While τ of 1000 is overestimated more than sixfold, the other τ values are underestimated up to threefold. Values of τ^n^ approximate model τ values of 33.3, 10 and 3.33. However, the τ^n^ value is overestimated 20,000 and 2.5 times for model values of 1000 and 100, respectively. Except for the τ value of 1000, operational efficacies calculated according to the Hill equation (Eq. (10)) are less than 10% off the model values.

### The case study

How fitting the Black & Leff equation to experimental data can affect estimates of the operational efficacy and subsequent analysis is demonstrated in the following example of measurement of the GTPγS binding as a functional response of M_2_ receptor to muscarinic agonists carbachol, iperoxo, N-desmethyl clozapine (NDMC) and oxotremorine at five subtypes of inhibitory G-proteins: G_i1_, G_i2_, G_i3_, G_oA_ and G_oB_ expressed in Sf9 cells. According to the saturation of [^3^H]NMS binding, Sf9 cell membranes contained about 7 pmol of binding sites per mg of membrane protein. Co-expression of individual subtypes of inhibitory G-proteins did not affect the expression level of M_2_ receptors. According to competition with [^3^H]NMS binding, tested agonists had the same affinity for M_2_ receptors in all co-expression systems (Supplementary Information Table S2).

When analysing the functional response, first, system E_MAX_ values were determined using the procedure described earlier^6^. They ranged from 84 to 88% of the maximum binding capacity of G-proteins. After subtraction of basal binding, responses were expressed as a fraction of E_MAX_. The system E_MAX_ was set equal to 1.

**Figure 6:**
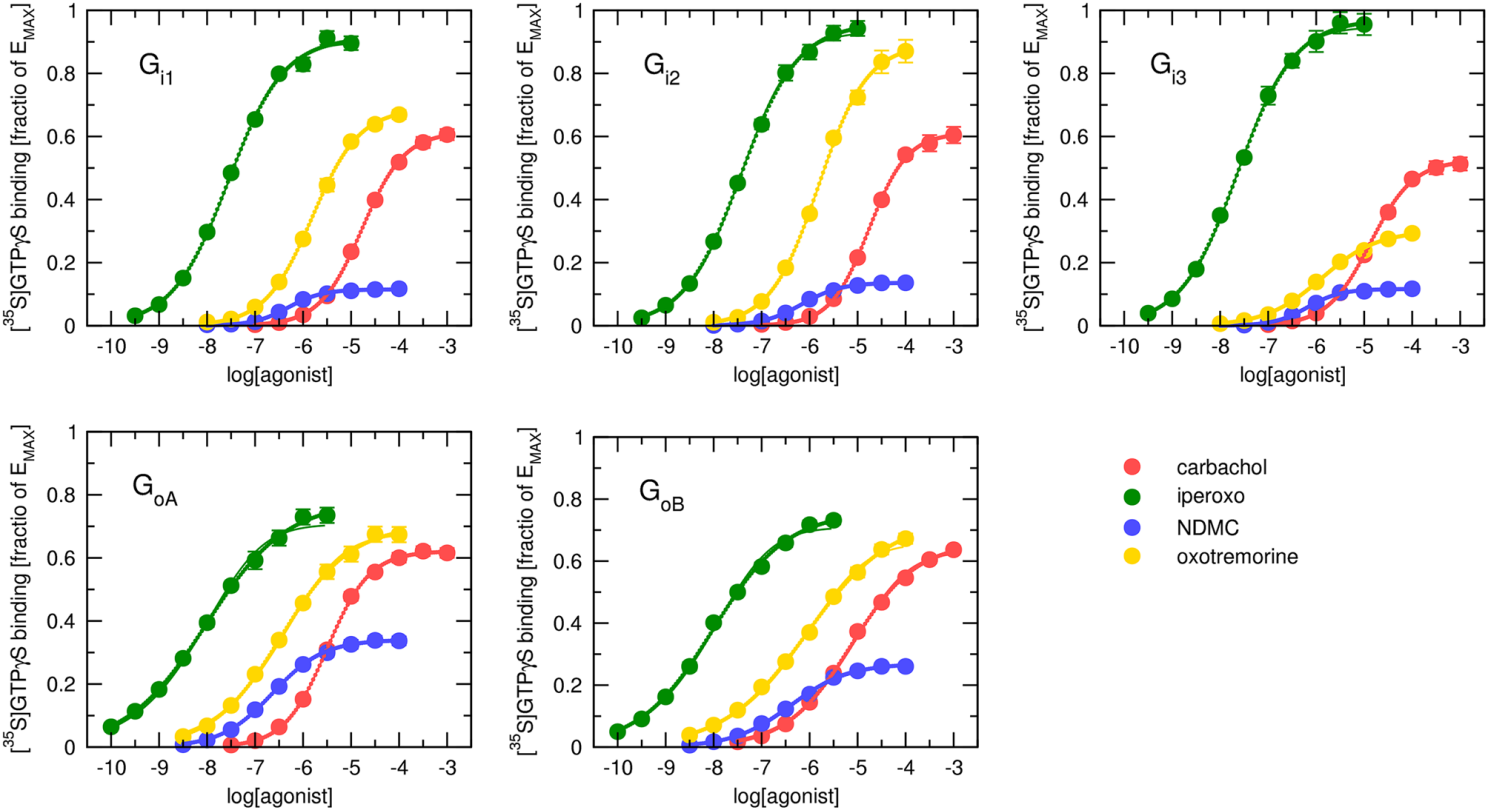
Functional response of muscarinic M_2_ receptors. GTPγS binding to G_i1_ (upper left), G_i2_ (upper middle), G_i3_ (upper right) G_oA_ (lower left) and G_oB_ (lower right) G-proteins upon stimulation of M_2_ muscarinic receptors by carbachol (red), iperoxo (green), NDMC (blue) or oxotremorine (yellow) at the concentration indicated on the x-axis is expressed as the fraction of maximal response of the system (E_MAX_). Data are means ± SD from 3 to 5 independent experiments performed in quadruplicates. Solid curves, Black & Leff equation (Eq. (7)). Dotted curves, Hill equation (Eq, (11)). Parameter estimates are in Supplementary Information Table S2.

Signalling profiles varied among subtypes of G-proteins. For example, carbachol and oxotremorine reached similar maximal responses E’_MAX_ at G_i1_, G_oA_ and G_oB_. At G_i2_, the E’_MAX_ of oxotremorine was substantially greater than the E’_MAX_ of carbachol. In contrast, at G_i3_, the E’_MAX_ of oxotremorine was substantially lower than the E’_MAX_ of carbachol (Fig. 6). Also, the steepness of functional responses to agonists varied among agonists as well as among subtypes of G-proteins. The functional response to carbachol was normal (n_H_ ≈ 1), except for a flat response (n_H_ = 0.68) at G_oB_ (Supplementary information Table S2). The functional response to NDMC was steep (n_H_ > 1.2) at G_i1_ and G_i3_, normal at G_i2_ and flat (n_H_ < 0.85) at G_oA_ and G_oB_. As the exact reason for variation in the steepness of functional-response curves is unknown, the Hill equation (Eq. (11)) was fitted to the experimental data for comparison with the fitting of the Black & Leff equation (Eq. (7)). Results of fitting are summarized in Supplementary Information Table S2).

**Figure 7:**
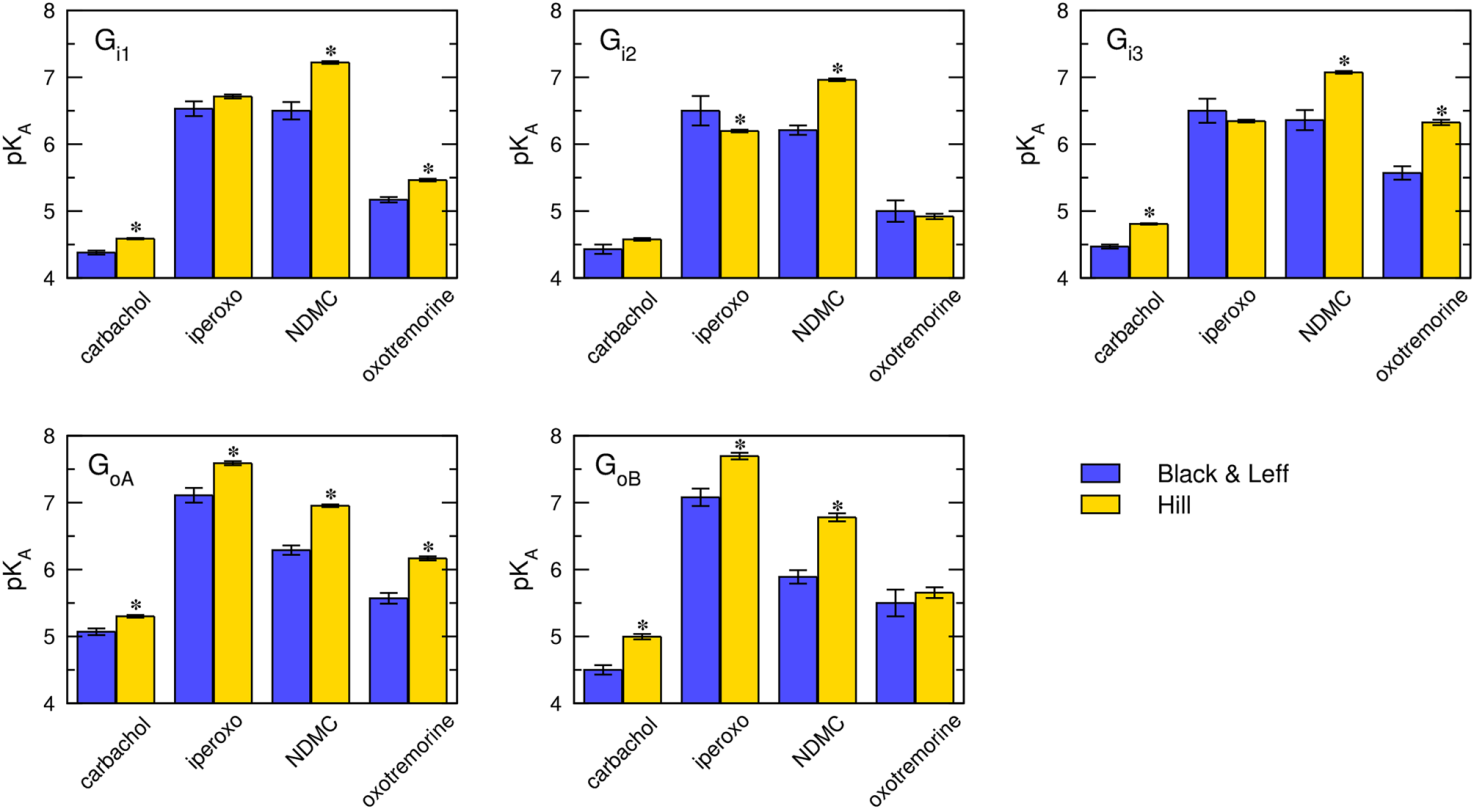
Analysis of pK_A_ estimates. Estimates of equilibrium dissociation constant K_A_ from fitting Black & Leff (Eq. (7)) (blue) or Hill (Eq. (11)) (yellow) equation are expressed as negative logarithms (pK_A_). Data are means ± SD from fits of 5 independent experiments performed in quadruplicates. *, different from Black & Leff (*p* < 0.05) according to ANOVA and Tukey HSD post-test.

The analysis of estimates of equilibrium dissociation constants K_A_ obtained from fitting Black & Leff (Eq. (7)) and Hill (Eq. (11)) equations shows that in comparison to Hill fits, estimates from Black & Leff fits are associated with greater variability (among individual fits) and uncertainty (individual fits) (Fig. 7). In the majority of cases, K_A_ estimates according to the Black & Leff equation were substantially lower than according to the Hill equation. Except for NDMC, K_A_ estimates for both models are higher than K_I_ from competition with [^3^H]NMS. Notably, agonist K_I_ values are the same for all subtypes of G-protein α-subunit while K_A_ values vary among them (Supplementary Information Table S2 and S3).

**Figure 8:**
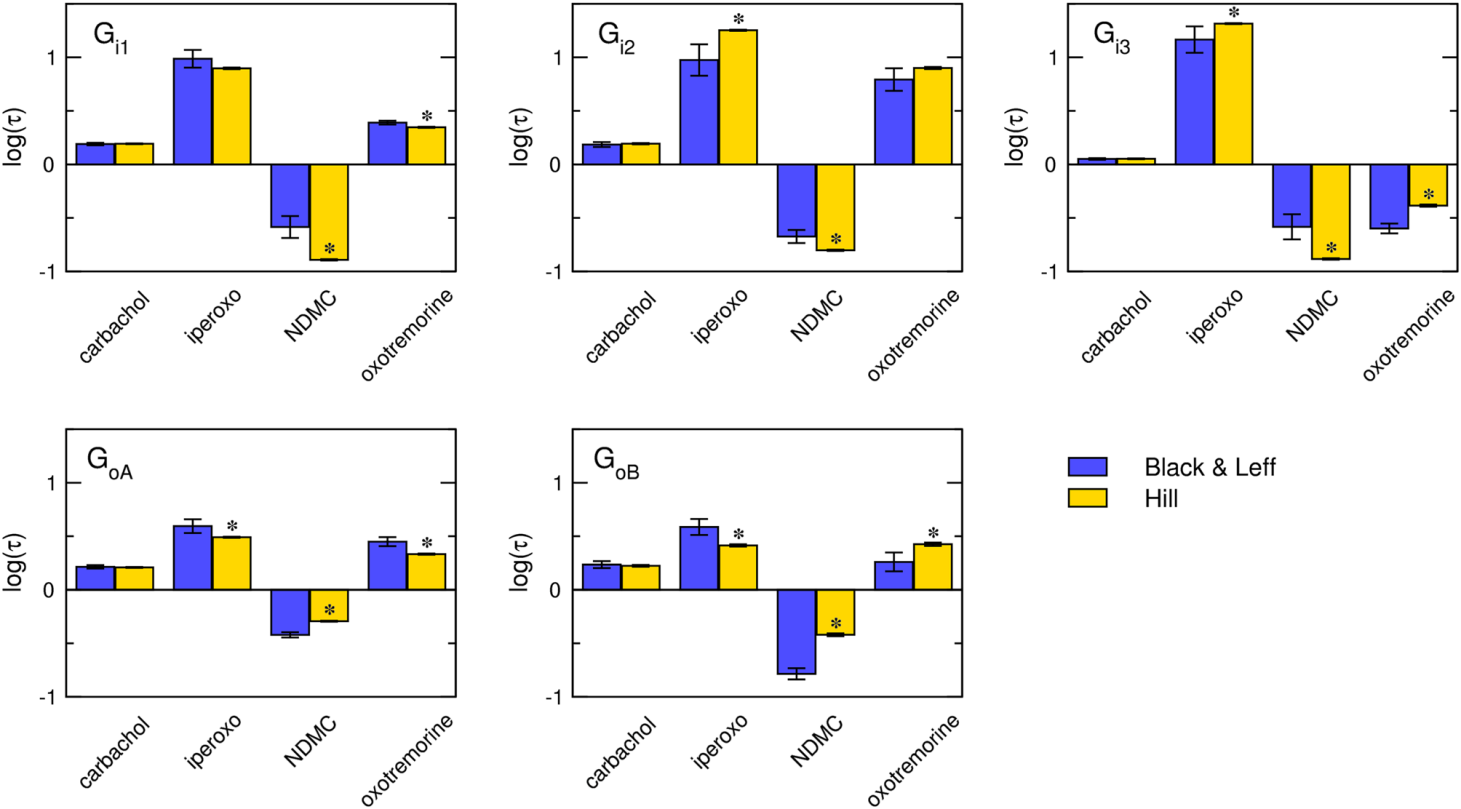
Analysis of τ estimates. Estimates of operational efficacy τ from fitting Black & Leff (Eq. (7)) (blue) or Hill (Eq. (11)) (yellow) equation are expressed as decadic logarithms (log τ). Data are means ± SD from fits of 5 independent experiments performed in quadruplicates. *, different from Black & Leff (*p* < 0.05) according to ANOVA and Tukey HSD post-test.

Similarly to estimates of K_A_, in comparison to Hill fits, estimates of operational efficacies τ from Black & Leff fits are associated with greater variability and uncertainty (Fig. 8). Overall, τ values tend to be underestimated for flat curves and overestimated for steep curves. Following analysis of the Black & Leff equation, this phenomenon is apparent for low operational efficacies, especially for τ < 1. In the extreme case of steep response to NDMC (n = 1.5) and flat response to oxotremorine (n = 0.7) at G_i3_, Black & Leff estimates of τ are the same for both agonists despite oxotremorine elicited E’_MAX_ three-times higher than NDMC. In the less extreme case, at G_oB_ G-protein, oxotremorine was about 60% more efficacious than carbachol but estimated τ values by Black & Leff were the same for both agonists although functional-response curves of both agonists are flat (n_H_ = 0.6 and 0.5, respectively). Thus, even a small change in the steepness of the functional-response curve has a profound effect on the estimation of the τ value.

## Discussion

The operational model of agonism (OMA)^2^ is widely used in the evaluation of agonism. The OMA characterizes a functional response to an agonist by the equilibrium dissociation constant of the agonist (K_A_), the maximal possible response of the system (E_MAX_) and the operational efficacy of the agonist (τ) (Eq. (4)). To fit non-hyperbolic functional responses slope factor **n** was introduced to the OMA (Eq. (7))^10^. Analysis of the Black & Leff equation (Eq. (7)) has shown that the slope factor **n** has a bidirectional effect on the relationship between the parameters E’_MAX_ and τ (Fig. 1A versus C) and also affects the relationship between the parameters EC_50_ and K_A_.

Fitting Black & Leff equation (Eq. (7)) to the models of non-competitive auto-inhibition (Fig. 3, Table 1), signalling feedback (Fig. 4, Table 2) and system with a similar expression of receptor and effector ([R_T_]≈[G_T_]) (Fig. 5, Table 3) resulted in inaccurate estimates of τ and K_A_ values. In the presented examples, the degree of over- or under-estimation of τ is not the same for all its values but depends on the value of τ, distorting relations among estimates of τ vales. In contrast, fitting the Hill equation (Eq. (10)) to the model data gave more or less accurate estimates of apparent operational efficacies τ’ from which operational efficacies can be calculated (according to Eq. (17), and Eq. (21)), provided that mechanism of action is known.

Biased agonists stabilize specific conformations of the receptor leading to non-uniform modulation of individual signalling pathways^27^. To measure an agonist bias, the parameters τ and K_A_ must be determined and log(τ/K_A_) values of tested and reference agonists compared at two signalling pathways^3^. It is evident from the analysis of the Black & Leff equation, that as far as the EC_50_ value is dependent on parameters **n** and τ (Fig. 1A,C), log(τ/K_A_) values cannot be compared to judge possible signalling bias unless the parameter **n** is equal to 1 for both tested and reference agonist.

Despite the dire effects of slope factor **n**, the Black & Leff equation is widely accepted^4,28–34^. It even entered textbooks^5^. Very little concern on factor **n** has risen. For example, Kenakin et al.^3^ analysed in detail the effects of slope factor **n** on EC_50_ and τ to K_A_ ratio but did not deal with the bi-directional effect of **n** on τ nor proposed an alternative approach to avoid potential pitfalls. To force a proper shape on functional-response curves whilst keeping slope factor **n** constant for all ligands, Gregory et al.^19^ introduced the second slope factor into their OMA and operational model of allosterically modulated agonism (OMAMA) analysis making equations even more complex. So far, the greatest criticism of OMA was voiced by Roche et al.^16^, noting that to accommodate the shape of theoretical curves Black & Leff equation tends to overestimate equilibrium dissociation constant K_A_ and operational efficacy τ and thus be misleading. They advocated for different expressions of operational models including OMA modified by the Hill coefficient in the case of symmetric concentration–response curves. Theoretical models show that in cases of non-competitive auto-inhibition and signal feedback where the slope of functional response does not vary, Black & Leff equation can be used to estimate apparent operational efficacy τ’ (Tables 1 and 2). However, estimates of operational efficacy do not approximate τ of the models.

The fitting of the Hill equation (Eq. (10)) to the functional response is straightforward and easier than fitting the Black & Leff equation. As shown in Figs. 3, 4, 5, 6, the Hill equation fits well with various functional-response curves, often better than the Black & Leff equation. In contrast to the Black & Leff equation, the Hill equation gives correct estimates of maximal response to agonist E’_MAX_ and its half-efficient concentration EC_50_ as documented in Tables. 1, 2, 3 and Figs. 7 and 8. In the case of the Hill equation, neither the value of E’_MAX_ nor the value of EC_50_ is affected by the Hill coefficient (Fig. 1B,D). Therefore, biased signalling may be inferred from the comparison of the ratio of intrinsic activity (E’_MAX_/EC_50_) of tested agonist to the intrinsic activity of reference agonist at two signalling pathways as in the case of the Hill equation the E’_MAX_/EC_50_ ratio is equivalent to τ/K_A_ ratio^35,36^. Further, if needed, apparent operational efficacy τ’ can be calculated from known E’_MAX_ values and known maximal response of the system E_MAX_. From the relationship between τ’ and EC_50_^6,17^, the mechanism of functional response can be inferred by comparison to explicit models.

The case study of the functional response of individual subtypes of inhibitory G-proteins to activation of the M_2_ muscarinic receptor (Fig. 6) demonstrated the pitfalls of exponentiation of operational efficacy τ: At G_i3_ G-protein, oxotremorine reached 3-times higher maximal response E’_MAX_ than NDMC. However, estimates of operational efficacy τ by the Black & Leff equation were the same for both agonists (Fig. 8), rendering it unsuitable for such data. An analysis of the Black & Leff equation implies that the source of the discrepancy lies in the profound effects of slope factor **n** on the estimation of τ value. The τ estimates by the Hill equation reflected differences in E’_MAX_ values, making it suitable for such a scenario. The root of the problem is variation in the slope of the functional response among tested agonists, indicating that the slope is not the property of the system required by the Black & Leff model. Rather agonists exert various degrees of cooperativity. As muscarinic receptors possess only one orthosteric binding site the data indicate that muscarinic receptors may function as oligomers^18^. Although cooperativity does not automatically mean oligomerization^37^, several lines of evidence indicate that muscarinic receptors may indeed oligomerize^38–41^.

## Conclusions

Analysis of the Black & Leff equation has shown that (i) The slope factor **n** has a bidirectional effect on the relationship between the parameters E’_MAX_ and τ. (ii) The slope factor **n** affects the relationship between the parameters EC_50_ and K_A_. Fitting the Black & Leff equation gives wrong estimates of τ and K_A_ values when slope factor **n** varies among concentration–response curves, limiting the use of the Black & Leff equation to evaluate concentration–response curves with the same slope. Analysis of the Hill equation has shown that the Hill coefficient does not affect the relationship between the parameters E’_MAX_ and τ nor between the parameters EC_50_ and K_A_. Fitting the Black & Leff equation to the experimental data demonstrated the drawbacks of exponentiation operational efficacy τ. In contrast, fitting the Hill equation to the experimental data gave more realistic estimates of K_A_ and τ. Black & Leff equation may be safely used only for systems where the slope of functional response does not vary substantially to estimate apparent operational efficacy.

## Methods

### Models and equations

Models and equations were derived from scratch as described in Supplementary Information. For modelling the theoretical curves and fitting curves to the experimental data the Python code employing numpy, scipy and matplotlib libraries was written.

### Preparation of cells and membranes

*Spodoptera frugiperda* cells (Sf9) (Gibco) were maintained as a suspension culture in serum-free insect cell growth medium SF900 III (Gibco) in a plastic Erlenmeyer flask in a shaking incubator at 27 °C and 135 rpm in a non-humidified environment. The cultures were maintained at a density of 1–4 × 10^6^ cells/ml. The density of the cells was determined with a haemocytometer, and viability was assessed by the exclusion of 0.2% trypan blue (Sigma-Aldrich). Human M_2_ receptors and α-subunits of (G_i1_, G_i2_, G_i3_, G_oA_ or G_oB_) G-proteins were expressed in recombinant baculoviruses, which were constructed and generated according to Bac-to-Bac® Baculovirus Expression System manual (Life Technologies)^42^.

One hundred ml of Sf9 cell suspension at a density of 2 × 10^6^ cells/ml were co-infected with baculoviruses encoding the M_2_ receptor and G_o_ or G_i_ α-subunit at a multiplicity of infection MOI = 0.1: 0.1. All infections were allowed to proceed for 69 h. Infected cells were harvested by centrifugation at 500 × g for 5 min and frozen at − 80 °C.

The pellets of harvested cells were suspended in the ice-cold homogenization medium (100 mM NaCl, 20 mM Na-HEPES, 10 mM EDTA, pH = 7.4) and homogenized on ice by two 30 s strokes using a Polytron homogenizer (Ultra-Turrax; Janke & Kunkel GmbH & Co. KG, IKA-Labortechnik, Staufen, Germany) with a 30-s pause between strokes. Cell homogenates were centrifuged for 5 min at 1000 × g. The supernatant was collected and centrifuged for 30 min at 30,000 × g. Pellets were suspended in the washing medium (100 mM NaCl, 10 mM MgCl_2_, 20 mM Na-HEPES, pH = 7.4), left for 30 min at 4 °C, and then centrifuged again for 30 min at 30,000 × g. The resulting membrane pellets were kept at − 80 °C until assayed.

### [^3^H]NMS binding

Membranes (approximately 10 μg of membrane proteins per sample) from Sf9 cells were incubated in 96-well plates for 3 (saturation) or 5 h (competition) at 25 °C in the incubation medium (100 mM NaCl, 20 mM Na-HEPES,10 mM MgCl_2_, pH = 7.4). The incubation volume for competition and saturation experiments with [^3^H]NMS was 400 μl or 800 μl, respectively.

In saturation experiments, eight concentrations of the [^3^H]NMS ranging from 94 to 1000 pM were used. Agonist binding was determined in competition experiments with 1 nM [^3^H]NMS. Nonspecific binding was determined in the presence of 10 μM unlabelled atropine. Incubation was terminated by filtration through Whatman GF/C glass fibre filters (Whatman) using a Brandel harvester (Brandel, USA). Filters were dried in a microwave oven (3 min, 800 W), and then solid scintillator Meltilex A was melted on filters (105 °C, 90 s) using a hot plate. The filters were cooled and counted in a Wallac Microbeta scintillation counter (Wallac, Finland).

### GTPγS binding

Agonist-stimulated [^35^S]GTPγS binding was performed as currently described^43^. Briefly, it was performed in 96-well plates at 30 °C in the incubation medium described above that was supplemented with freshly prepared dithiothreitol at a final concentration of 1 mM. Suspension of membranes of Sf9 cells expressing M_2_ + G-protein α-subunit were preincubated with GDP and agonists for 15 min, then [^35^S]GTPγS was added for an additional 20 min. The final concentration of GDP and [^35^S]GTPγS was 20 µM and 500 pM, respectively. The maximum binding capacity of G-proteins was determined in the absence of GDP. Nonspecific binding was determined in the presence of 1 µM non-labelled GTPγS. Incubations were terminated by filtration through GF/C filtration plates (Millipore) using a Brandel cell harvester (Perkin Elmer, USA). Plates were dried in a microwave oven at 800W for 3 min and then 40µl of ROTISZINT® Eco Plus (ROTH) was added. The plates were counted in the Wallac Microbeta scintillation counter.

### Analysis of experimental data

Experimental data were analysed using the two-step procedure described earlier^6,17^. First, the maximum system response E_MAX_ was determined. After subtracting the value of the basal signal, functional responses were expressed as the fraction of corresponding E_MAX_. Then, the Hill equation (Eq. (10)) and the Black & Leff equation (Eq. (7)) were fitted to the data. In the case of the Hill equation, the maximum response to an agonist E’_MAX_ was confined to less than 1. In the case of the Black & Leff equation, system E_MAX_ was set equal to 1.

### Supplementary Information

Supplementary Information.

## Data Availability

The data that support the findings of this study are available from the corresponding author upon reasonable request.

## References

[1] Stephenson RP (1956). A modification of receptor theory. Br. J. Pharmacol. Chemother..

[2] Black JW, Leff P (1983). Operational models of pharmacological agonism. Proc. R. Soc. London. Ser. B Biol. Sci..

[3] Kenakin T, Watson C, Muniz-Medina V, Christopoulos A, Novick S (2012). A simple method for quantifying functional selectivity and agonist bias. ACS Chem. Neurosci..

[4] Kenakin T, Christopoulos A (2013). Signalling bias in new drug discovery: detection, quantification and therapeutic impact. Nat. Rev. Drug Discov..

[5] Kenakin, T. P. Agonists: The Measurement of Affinity and Efficacy in Functional Assays. In *A Pharmacology Primer* 85–117 (Academic Press, 2014). doi:10.1016/b978-0-12-407663-1.00005-3.

[6] Jakubík J (2019). Applications and limitations of fitting of the operational model to determine relative efficacies of agonists. Sci. Rep..

[7] Hall DA, Giraldo J (2018). A method for the quantification of biased signalling at constitutively active receptors. Br. J. Pharmacol..

[8] Onaran HO (2017). Systematic errors in detecting biased agonism: Analysis of current methods and development of a new model-free approach. Sci. Rep..

[9] Onaran HO, Costa T (2021). Conceptual and experimental issues in biased agonism. Cell. Signal..

[10] Black JW, Leff P, Shankley NP, Wood J (1985). An operational model of pharmacological agonism: the effect of E/[A] curve shape on agonist dissociation constant estimation. Br. J. Pharmacol..

[11] Hill AV (1910). The possible effects of the aggregation of the molecules of hæmoglobin on its dissociation curves. J. Physiol..

[12] Gesztelyi R (2012). The Hill equation and the origin of quantitative pharmacology. Arch. Hist. Exact Sci..

[13] Clark AJ (1926). The antagonism of acetyl choline by atropine. J. Physiol..

[14] Furchgott RF (1966). The use of β-haloalkylamines in the diferentiation of receptors and in the determination of dissociation constants of receptor-agonist complexes. Adv. Drug Res..

[15] Weiss JN (1997). The Hill equation revisited: Uses and misuses. FASEB J..

[16] Roche D, van der Graaf PH, Giraldo J (2016). Have many estimates of efficacy and affinity been misled? Revisiting the operational model of agonism. Drug Discov. Today.

[17] Jakubík J, Randáková A, Chetverikov N, El-Fakahany EE, Doležal V (2020). The operational model of allosteric modulation of pharmacological agonism. Sci. Rep..

[18] Jakubík J, Randáková A (2022). Insights into the operational model of agonism of receptor dimers. Exp. Opin. Drug Discov..

[19] Gregory KJ, Giraldo J, Diao J, Christopoulos A, Leach K (2020). Evaluation of operational models of agonism and allosterism at receptors with multiple orthosteric binding sites. Mol. Pharmacol..

[20] Strelow, J. *et al. Mechanism of Action Assays for Enzymes*. *Assay Guidance Manual* (2004).

[21] Ogawa H, Sato M, Yamashita S (1969). Gustatory impulse discharges in response to saccharin in rats and hamsters. J. Physiol..

[22] Alberts P, Bartfai T, Stjärne L (1981). Site(s) and ionic basis of alpha-autoinhibition and facilitation of "3H’noradrenaline secretion in guinea-pig vas deferens. J. Physiol..

[23] Li S-J (2016). Cooperative autoinhibition and multi-level activation mechanisms of calcineurin. Cell Res..

[24] Del Vecchio, D. & Murray, R. M. *Biomolecular Feedback Systems*. *Biomolecular Feedback Systems* (Princeton University Press, 2014). doi:10.23943/princeton/9780691161532.001.0001.

[25] Black JB, Premont RT, Daaka Y (2016). Feedback regulation of G protein-coupled receptor signaling by GRKs and arrestins. Semin. Cell Dev. Biol..

[26] Gómez-Schiavon M, El-Samad H (2022). CoRa-A general approach for quantifying biological feedback control. Proc. Natl. Acad. Sci. U. S. A..

[27] Lefkowitz RJ (2013). A brief history of G-protein coupled receptors (Nobel Lecture). Angew. Chem. Int. Ed. Engl..

[28] Christopoulos A, El-Fakahany EE (1999). Qualitative and quantitative assessment of relative agonist efficacy. Biochem. Pharmacol..

[29] Kenakin TP (2012). Biased signalling and allosteric machines: New vistas and challenges for drug discovery. Br. J. Pharmacol..

[30] Keov P (2014). Molecular mechanisms of bitopic ligand engagement with the M1 muscarinic acetylcholine receptor. J. Biol. Chem..

[31] Luttrell LM, Maudsley S, Bohn LM (2015). Fulfilling the promise of ‘biased’ g protein-coupled receptor agonism. Mol. Pharmacol..

[32] Stott LA, Hall DA, Holliday ND (2016). Unravelling intrinsic efficacy and ligand bias at G protein coupled receptors: A practical guide to assessing functional data. Biochem. Pharmacol..

[33] Burgueño J (2017). A complementary scale of biased agonism for agonists with differing maximal responses. Sci. Rep..

[34] Kenakin T (2017). A scale of agonism and allosteric modulation for assessment of selectivity, bias, and receptor mutation. Mol. Pharmacol..

[35] Ehlert FJ, Griffin MT, Sawyer GW, Bailon R (1999). A simple method for estimation of agonist activity at receptor subtypes: Comparison of native and cloned M3 muscarinic receptors in guinea pig ileum and transfected cells. J. Pharmacol. Exp. Ther..

[36] Griffin MT, Figueroa KW, Liller S, Ehlert FJ (2007). Estimation of agonist activity at G protein-coupled receptors: Analysis of M2 muscarinic receptor signaling through Gi/o, Gs, and G15. J. Pharmacol. Exp. Ther..

[37] Chabre M, Deterre P, Antonny B (2009). The apparent cooperativity of some GPCRs does not necessarily imply dimerization. Trends Pharmacol. Sci..

[38] Park PSH, Wells JW (2004). Oligomeric potential of the M2 muscarinic cholinergic receptor. J. Neurochem..

[39] Hu J (2012). Structural aspects of M_3_ muscarinic acetylcholine receptor dimer formation and activation. FASEB J..

[40] Redka DS (2014). Coupling of G proteins to reconstituted monomers and tetramers of the M2 muscarinic receptor. J. Biol. Chem..

[41] Liste MJV (2015). The molecular basis of oligomeric organization of the human M3 muscarinic acetylcholine receptor. Mol. Pharmacol..

[42] Anderson D (1995). Rapid generation of recombinant baculovirus and expression of foreign genes using the BAC-to-BAC Baculovirus expression system. Focus Madison..

[43] Randáková A (2020). Agonist-specific conformations of the M2 muscarinic acetylcholine receptor assessed by molecular dynamics. J. Chem. Inf. Model..

