# Supplementary material for: A critical re-evaluation of the slope factor of the operational model of agonism: When to exponentiate operational efficacy
Source: Sci Rep. 2023 Oct 16;13:17587. doi: 10.1038/s41598-023-45004-7 (PMC10579308; doi:10.1038/s41598-023-45004-7)
Supplement: Supplementary file 1 — Supplementary Information. [file 41598_2023_45004_MOESM1_ESM.pdf]

Supplementary Information: A critical re-evaluation of the slope factor of the operational model of agonism: When to exponentiate operational efficacy

Short title: On exponentiation of operational efficacy

Alena Randáková, Dominik Nelic, Jan Jakubík*
Institute of Physiology Czech Academy of Sciences, Prague, Czech Republic

*, Correspondence: Jan Jakubik, Inst. of Physiology CAS, Vídeňská 1083, 142 20 Praha, Czech Republic, jan.jakubik@fgu.cas.cz

Hyperbola

A hyperbola is a set of points in a plane whose distances from two fixed points, called foci, has an absolute difference that is equal to a positive constant. It consists of two separate curves, called branches. Hyperbola is completely determined by its centre, vertices, and asymptotes. Points on the separate branches of the graph where the distance is at a minimum are called vertices. The midpoint between vertices is its centre. Hyperbola is asymptotic to certain lines drawn through the centre. Hyperbola which asymptotes form the right angle is called rectangular or equilateral. The simplest rectangular hyperbola with coordinate axes as its asymptotes is reciprocal function y = 1/x (Figure S1, black). The reciprocal function can be geometrically translated by coefficients a, b, c, and d (Figure S1, green a=1, b=1, c=1, d=-1).

Eq. S1

$$y = \frac{ax+b}{cx+d}$$

Where vertical and horizontal asymptotes are given by Eq. A2 and A3, respectively.

Eq. S2

$$\lim_{y\to\pm\infty} = -\frac{d}{c}$$

Eq. S3

$$\lim_{x\to\pm\infty} = \frac{a}{c}$$

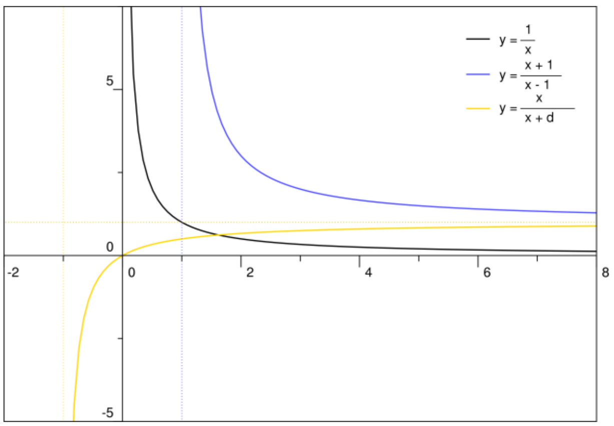

Figure S1 Examples of rectangular hyperbolas

Green, hyperbola with parameters a=1, b=1, c=1 and d=-1. Red, hyperbola with parameters a=1, b=0, c=1, d=1. Dashed lines, vertical and horizontal asymptotes of respective hyperbolas. Only the right branches are shown.

A hyperbola with parameters a > 0, b = 0, c = 1 and d > 0 (e.g., Figure S1 red) is a mass action equilibrium function.

Power function

Exponentiation is a mathematical operation, written as b^n, involving two numbers, the base b and the exponent n. $b \geq 0$ can be risen to any value of n. $b < 0$ can be risen only to integers or fractions with odd denominators. Table S1 summarizes relationships between b^n and b values ≥ 0. Reciprocal relationships apply for b values < 0 as the power function is symmetric at the origin of axes. Importantly relationship between values of b^n and n are opposite in the range $0 < b < 1$ to the range $b > 1$, resulting in the S-shape of the function (Figure S2).

Table S1 Behaviour of power function, the relationship between b^n and b values.

	**n = 0**	**0 < n < 1**	**n = 1**	**n >1**
**b = 0**	$b^n = 1$	$b^n = b$	$b^n = b$	$b^n = b$
**0 < b <1**	$b^n = 1$	$b^n > b$	$b^n = b$	$b^n < b$
**b = 1**	$b^n = 1$	$b^n = b$	$b^n = b$	$b^n = b$
**b > 1**	$b^n = 1$	$b^n < b$	$b^n = b$	$b^n > b$

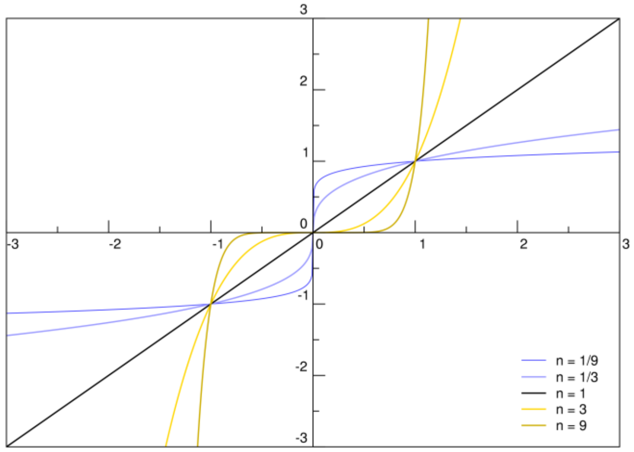

Figure S2 Examples of power functions

Curves of $y = x^n$ for various values of exponent n. Each curve passes through the point (0, 0) because 0 raised to any power is 0 and through the point (1, 1) because the number 1 raised to any power is 1. For n = 1, y = x because any number raised to the power of 1 is the number itself.

Hyperbola and power function

Exponentiation of x in the hyperbola (Eq. A1) results in a non-hyperbolic function (Eq. A4, Figure S3).

Eq. S4

$$y = \frac{ax^n+b}{cx^n+d}$$

However, the resulting function still possesses asymptotes given by Eq. A2 and A3 (Figure S3).

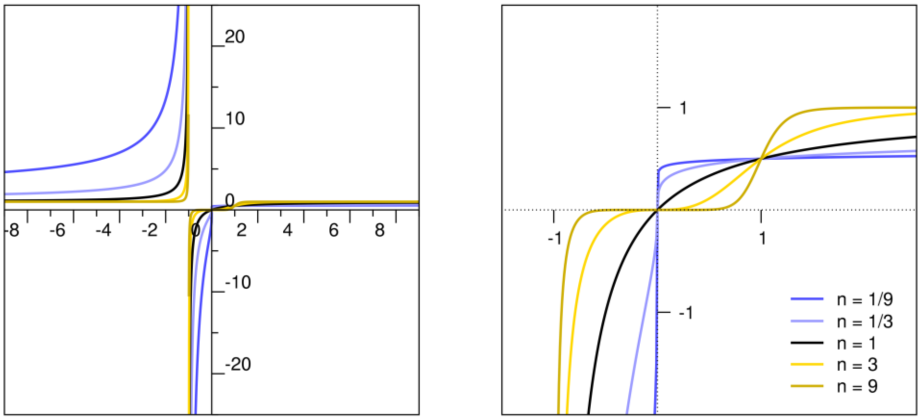

Figure S3 Examples of functions according to Eq. A4

Curves according to Eq. A4 for various values of exponent n. Left, both branches are shown. Right, detail of right branches. Black, for the n=1, the curve is a rectangular hyperbola. Shades of blue, n < 1, and shades of yellow, n > 1, curves are S-shaped.

OMA from scratch

Binding:

Eq. S5

$$[RA] = \frac{[R_T][A]}{K_A+[A]}$$

Functional response:

Eq. S6

$$E = E_{MAX}\frac{[RA]}{K_E+[RA]}$$

Substitution of [RA] by Eq. S5:

Eq. S7

$$E = E_{MAX}\frac{\frac{[R_T][A]}{K_A+[A]}}{K_E+\frac{[R_T][A]}{K_A+[A]}}$$

After rearrangement:

Eq. S8

$$E = E_{MAX}\frac{[R_T][A]}{[A](K_E+[R_T])+K_EK_A}$$

Division by K_E and substitution $\tau=[R_T]/K_E$:

Eq. S9

$$E = E_{MAX}\frac{\tau[A]}{[A](1+\tau)+K_A}$$

Apparent E'_{MAX} for $[A]>>K_A$:

Eq. S10

$$E'_{MAX} = E_{MAX}\frac{\tau}{1+\tau}$$

For half-efficient concentration EC_{50} Eq. S9 equals half of Eq. S10:

Eq. S11

$$E_{MAX}\frac{\tau EC_{50}}{EC_{50}(1+\tau)+K_A} = \frac{E_{MAX}}{2}\frac{\tau}{1+\tau}$$

Eq. S12

$$\frac{EC_{50}}{EC_{50}(1+\tau)+K_A} = \frac{1}{2(1+\tau)}$$

Eq. S13

$$\frac{EC_{50}2(1+\tau)}{(1+\tau)\left(EC_{50}+\frac{K_A}{1+\tau}\right)} = 1$$

Eq. S14

$$2EC_{50} = EC_{50}+\frac{K_A}{1+\tau}$$

Eq. S15

$$EC_{50} = \frac{K_A}{1+\tau}$$

Non-competitive auto-inhibition

In the case of non-competitive auto-inhibition, the functional response is given by Eq. S16.

Eq. S16

$$E = E_{MAX}\frac{[RA]}{K_E+[RA]}\frac{[RA]}{K_I+[RA]}$$

Substitution of the [RA] complexes in Eq. S16 with binding function Eq. S5 yields Eq. S17.

Eq. S17

$$E = E_{MAX}\frac{\frac{[R_T][A]}{K_A+[A]}}{K_E+\frac{[R_T][A]}{K_A+[A]}}\frac{\frac{[R_T][A]}{K_A+[A]}}{K_I+\frac{[R_T][A]}{K_A+[A]}}$$

That simplifies into Eq. S18.

Eq. S18

$$E = E_{MAX}\frac{[R_T][A]}{K_AK_E+K_E[A]+[R_T][A]}\frac{[R_T][A]}{K_AK_I+K_I[A]+[R_T][A]}$$

Division of Eq. S18 by K_E and K_I and substitution $\tau = [R_T]/K_E$ and $\sigma = [R_T]/K_I$ gives Eq. S19.

Eq. S19

$$E = E_{MAX}\frac{\tau[A]}{K_A+[A](1+\tau)}\frac{\sigma[A]}{K_A+[A](1+\sigma)}$$

Apparent E'_{MAX} at [A]>>K_A is given by Eq. S20.

Eq. S20

$$E'_{MAX} = E_{MAX} \frac{\tau}{1+\tau} \frac{\sigma}{1+\sigma}$$

The apparent value of operational efficacy τ' is given by equation Eq. S21.

Eq. S21

$$\tau' = \frac{\tau\sigma}{\tau+\sigma+1}$$

For half-efficient concentration EC_{50}:

Eq. S22

$$\frac{1}{2} E_{MAX} \frac{\tau}{1+\tau} \frac{\sigma}{1+\sigma} = E_{MAX} \frac{\tau EC_{50}}{K_A+EC_{50}(1+\tau)} \frac{\sigma EC_{50}}{K_A+EC_{50}(1+\sigma)}$$

Eq. S23

$$\frac{1}{2} = \frac{EC_{50}(1+\tau)}{K_A+EC_{50}(1+\tau)} \frac{EC_{50}(1+\sigma)}{K_A+EC_{50}(1+\sigma)}$$

Eq. S24

$$EC_{50} = K_A \frac{\sqrt{\sigma^2+6\sigma\tau+8\sigma+\tau^2+8\tau+8}+\sigma+\tau+2}{2(1+\sigma)(1+\tau)}$$

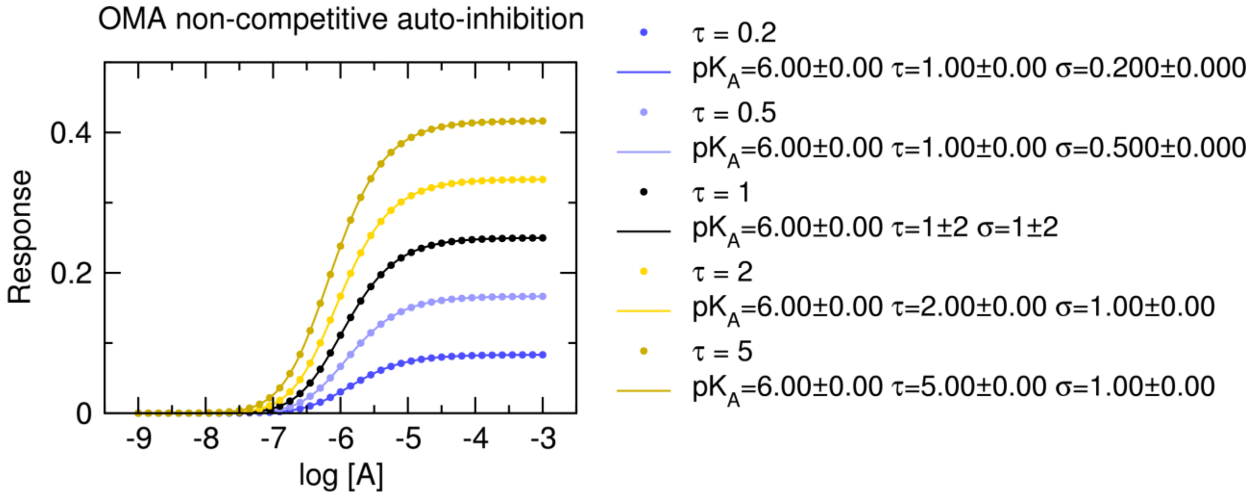

Figure S4 Fitting Eq. S19 to the model of non-competitive auto-inhibition

Dots, functional-response data modelled according to Eq. S17. E_{MAX} = 1, K_A = 10^{-6}M, σ = 1, R_T =1. Values of operational efficacy τ are indicated in the legend. Lines, fits of Eq. S19 to the model data. E_{MAX} was fixed to 1, and the initial estimate of τ was set to 0.3. Parameter estimates of the fits are indicated in the legend.

Except for τ = 1, parameter estimates are correct, and associated with the low level of uncertainty. However, it is the result of the initial estimate of τ. For τ = 1 = σ, estimates are also correct but associated with a large uncertainty level as so far τ = σ curves given by Eq. S19 do not change.

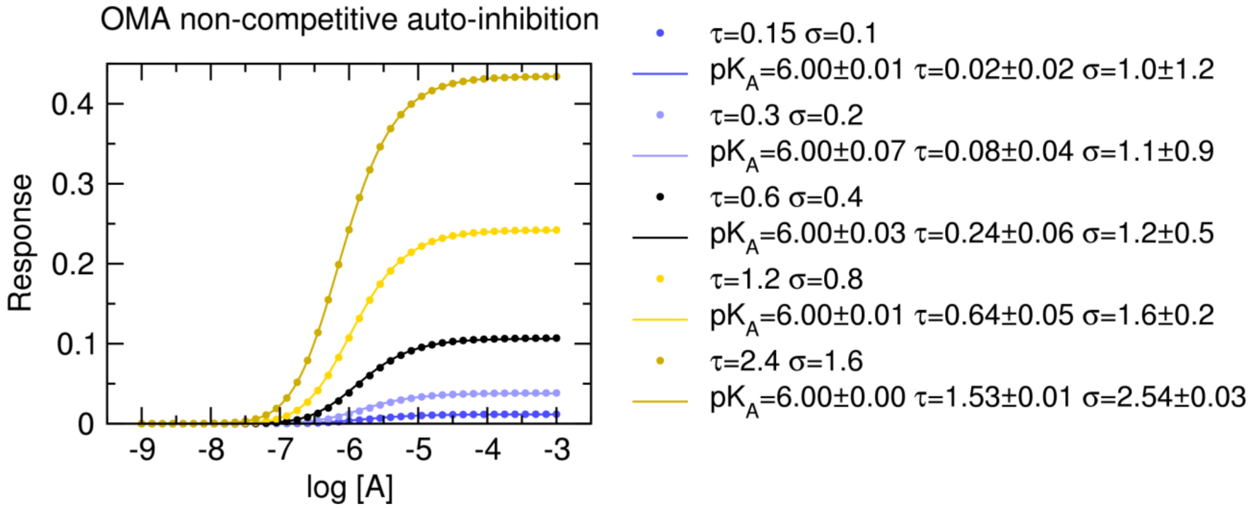

Figure S5 Fitting Eq. S19 to the model of non-competitive auto-inhibition with varying receptor concentration

Dots, functional-response data modelled according to Eq. S17. E_{MAX} = 1, K_A = 10^{-6}M, K_E = 3.333, K_I = 5. R_T varied from 0.5 to 8. The resulting operational efficacies τ and inhibition factors σ are indicated in the legend. Lines, fits of Eq. S19 to the model data. E_{MAX} was fixed to 1, and the initial estimate of τ was set to apparent operational efficacy τ' calculated as $E'_{MAX}/(E_{MAX}-E'_{MAX})$. Parameter estimates of the fits are indicated in the legend.

Parameter estimates are incorrect and for low values of τ and σ they are associated with high-level uncertainty. However, calculated τ and σ values give correct apparent efficacy according to Eq. S21.

Signalling feedback

In signalling feedback, an increase in output signal proportionally either decreases (negative feedback) or increases (positive feedback) input. Activation constant K_E can be expressed as the difference between [RAG] formation and decay Eq. S 25.

Eq. S 25

$$K_E = \frac{[G_T][RA]}{[RAG]} - \frac{[RA][RAG]}{[RAG]}$$

Where $[G_T]$ is the total concentration of the effector. Eq. S 25 can be simplified to Eq. S 26.

Eq. S 26

$$K_E = \frac{[G_T][RA]}{[RAG]} - [RA]$$

After rearrangement

Eq. S 27

$$[RAG] = \frac{[G_T][RA]}{K_E+[RA]}$$

The feedback factor δ modifies input [RA]

Eq. S 28

$$K_E = \frac{[G_T][RA]}{[RAG]} - \delta[RA]$$

Respectively

Eq. S 29

$$[RAG] = \frac{[G_T][RA]}{K_E+\delta[RA]}$$

For δ > 1, the right member of Eq. S 28 is greater than the right member of Eq. S 26 and thus denotes attenuation of the signal, negative feedback. Conversely, δ < 1 denotes positive feedback. The proportion of signal output, [RAG], working as feedback is constant and is given by the division of Eq. S 29 by Eq. S 27.

Eq. S 30

$$\frac{K_E+[RA]}{K_E+\delta[RA]}$$

Under the feedback, the response is given by:

Eq. S 31

$$[RAG] = \frac{[G_T][RA]\frac{K_E+[RA]}{K_E+\delta[RA]}}{K_E+[RA]\frac{K_E+[RA]}{K_E+\delta[RA]}}$$

After simplification

Eq. S 32

$$[RAG] = \frac{[G_T][RA]}{[RA]+K_E\frac{K_E+\delta[RA]}{K_E+[RA]}}$$

Taking [RAG] as a functional response E and [E_T] as the maximal response of the system E_{MAX} and substitution binding equation (Eq. S5) into Eq. S 32 we receive:

Eq. S 33

$$E = E_{MAX}\frac{\frac{[R_T][A]}{K_A+[A]}}{\frac{[R_T][A]}{K_A+[A]}+K_E\frac{K_E+\delta\frac{[R_T][A]}{K_A+[A]}}{K_E+\frac{[R_T][A]}{K_A+[A]}}}$$

After simplification:

Eq. S 34

$$E = E_{MAX}\frac{[R_T][A]([A]K_E+[A][R_T]+K_AK_E)}{[A]^2[R_T]^2+[A](\delta+1)K_E[R_T]([A]+K_A)+K_E^2([A]+K_A)^2}$$

After the multiplication of parentheses:

Eq. S 35

$$E = E_{MAX}\frac{[R_T][A]^2K_E+[A]^2[R_T]^2+[R_T][A]K_AK_E}{\delta[A]^2K_E[R_T]+[A]^2K_A^2+[A]^2K_E[R_T]+[A]^2[R_T]^2+\delta[A]K_AK_E[R_T]+2[A]K_AK_E^2+[A]K_AK_E[R_T]+K_A^2K_E^2}$$

After division by K_E^2 and substitution $\tau = [R_T]/K_E$:

Eq. S 36

$$E = E_{MAX}\frac{\tau[A]^2+\tau^2[A]^2+\tau[A]K_A}{\delta\tau[A]^2+[A]^2+\tau[A]^2+\tau^2[A]^2+\delta\tau[A]K_A+2[A]K_A+\tau[A]K_A+K_A^2}$$

After simplification:

Eq. S 37

$$E = E_{MAX}\frac{\tau[A](\tau[A]+[A]+K_A)}{[A]^2(\delta\tau+\tau^2+\tau+1)+[A]K_A(\delta\tau+\tau+2)+K_A^2}$$

For $[A]>>K_A$ Eq. S 37 becomes:

Eq. S 38

$$E = E_{MAX}\frac{\tau[A](\tau[A]+[A])}{[A]^2(\delta\tau+\tau^2+\tau+1)}$$

Thus, the maximal response to an agonist with operational efficacy τ is:

Eq. S 39

$$E'_{MAX} = E_{MAX}\frac{\tau^2+\tau}{\delta\tau+\tau^2+\tau+1}$$

And apparent value of operational efficacy is:

Eq. S 40

$$\tau' = \frac{\tau^2+\tau}{\delta\tau+1}$$

Solving Eq. S 37 for ½ E'_{MAX} gives EC_{50} value as:

Eq. S 41

$$EC_{50} = \frac{1}{2}\sqrt{\frac{K_A^2((\delta^2-2\delta+5)\tau^4-2(\delta^2-4\delta-5)\tau^3+(\delta^2+6\delta+17)\tau^2+4(\delta+3)\tau+4)}{(\tau+1)^2(\delta\tau+\tau^2+\tau+1)^2}}+\frac{(\delta-1)K_A(\tau-1)\tau}{2(\tau+1)(\delta\tau+\tau^2+\tau+1)}$$

Alternatively,

Eq. S 42

$$EC_{50} = K_A\frac{\sqrt{(\delta(\delta-2)+5)\tau^4-2(\delta-5)(\delta+1)\tau^3+(\delta(\delta+6)+17)\tau^2+4(\delta+3)\tau+4}+(\delta-1)(\tau-1)\tau}{2(\tau+1)(\tau(\delta+\tau+1)+1)}$$

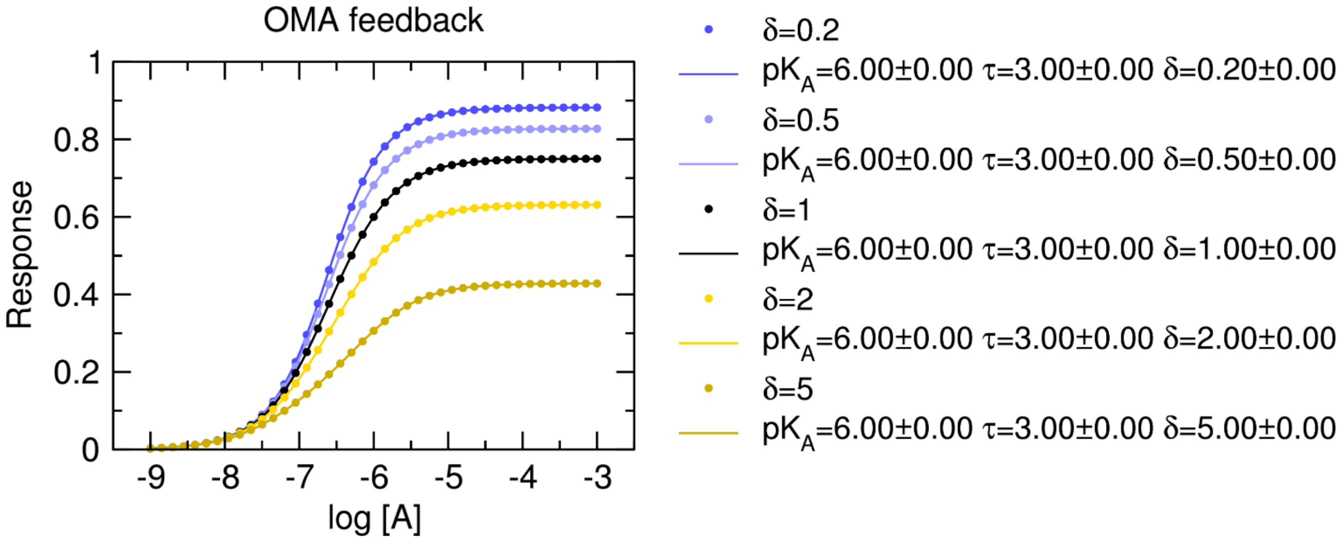

Figure S6 Fitting Eq. S 37 to the signalling-feedback model with varying feedback

Dots, functional-response data modelled according to Eq. S 33. E_{MAX} = 1, K_A = 10^{-6}M, K_E = 0.333, R_T = 1. Feedback factor δ varied from 0.2 to 5 and is indicated in the legend. Lines, fits of Eq. S 37 to the model data. E_{MAX} was fixed to 1, the initial estimate of τ was set to 3 and the initial estimate of pK_A was set to 6. Parameter estimates of the fits are indicated in the legend.

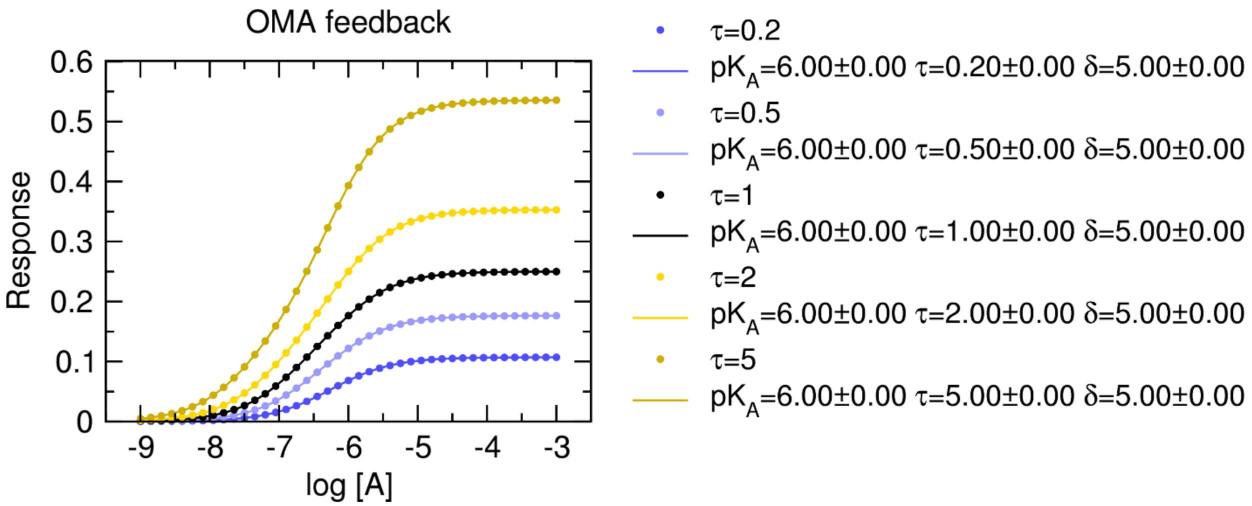

Figure S7 Fitting Eq. S 37 to the signalling-feedback model with constant feedback and varying operational efficacy

Dots, functional-response data modelled according to Eq. S 33. E_{MAX} = 1, K_A = 10^{-6}M, R_T = 1, δ=5. Operational efficacy varied from 0.2 to 5 and is indicated in the legend. Lines, fits of Eq. S 37 to the model data. E_{MAX} was fixed to 1, the initial estimate of δ was set to 5 and the initial estimate of pK_A was set to 6. Parameter estimates of the fits are indicated in the legend.

The system with a similar expression of receptor and effector ($[R_T]\approx[G_T]$)

Equation Eq. S6 is valid only when [RA]>>$[G_T]$ or [RA]<<$[G_T]$. For $[R_T]\approx[G_T]$ K_E is given by:

Eq. S43

$$K_E = \frac{[RA_F][G_F]}{[RAG]}$$

Where [RA_F] and [G_F] are free concentrations of receptor-agonist complexes RA and effector G, respectively, and are given by:

Eq. S44

$$[RA_F] = [RA] - [RAG]$$

Eq. S45

$$[G_F] = [G_T] - [RAG]$$

Where [RA] and [G_T] are total concentrations of RA and G, respectively, and RAG is a complex of RA and G. By substitution Eq. S44 and Eq. S45 into Eq. S43:

Eq. S46

$$K_E = \frac{([RA]-[RAG])([G_T]-[RAG])}{[RAG]}$$

Rearranging:

Eq. S47

$$K_E\,[RAG] = [RA][G_T] - [RA_F][RAG] - [RAG][G_T] + [RAG]^2$$

Eq. S48

$$K_E = \frac{[RA][G_T]}{[RAG]} - [RA] - [G_T] + [RAG]$$

Eq. S49

$$\frac{[RA][G_T]}{[RAG]} - [RA] - [G_T] + [RAG] - K_E = 0$$

Solving Eq. S49 for [RAG] gives:

Eq. S50

$$[RAG] = \frac{1}{2}\left(K_E + [RA] + [G_T] - \sqrt{K_E^2 + 2K_E([RA] + [G_T]) + ([RA] - [G_T])^2}\right)$$

Substituting binding function (Eq. S5) for [RA_T] into Eq. S49 gives:

Eq. S51

$$\frac{\frac{[R_T][A]}{K_A+[A]}[G_T]}{[RAG]} - \frac{[R_T][A]}{K_A+[A]} - [G_T] + [RAG] - K_E = 0$$

That simplifies to:

Eq. S52

$$[RAG] - K_E - [G_T] - \frac{[A][G_T][R_T]([RAG]-1)}{[RAG]([A]+K_A)} = 0$$

Solving Eq. S52 for [RAG] gives only approximate solutions.

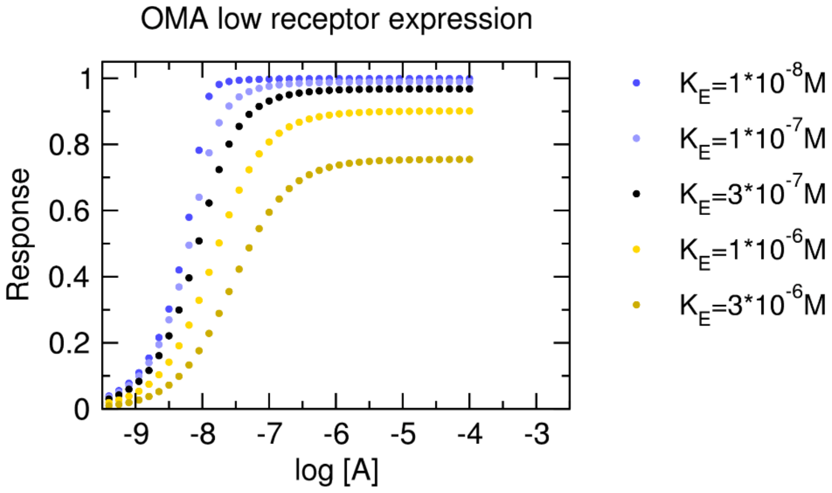

Figure S 8 Modelling the system with a similar expression of receptor and effector ($[R_T] \approx [G_T]$)

Dots, functional-response data modelled in two steps. First, binding was calculated according to Eq. S5. Then resulting [RA] was used in Eq. S50. $G_T = 10^{-6}$M, $R_T = 10^{-5}$M, $K_A = 10^{-6}$M. Values of K_E are indicated in the legend. As Eq. S52 gives only approximate solutions, data were not refitted.

The case study

Stimulation of [^{35}S]GTPγS binding to subtypes of inhibitory G-proteins upon activation of the M_2 muscarinic receptor by agonists.

Table S2 Inhibition constant K_I of tested agonist

Inhibition constants (K_I) were determined from competition with [3H]NMS binding to membranes from Sf9 cell co-expressing M_2 receptor and individual isoforms of G-protein α-subunit. Values are expressed as means ± SD of negative logarithms (pK_I) of 3 independent experiments performed in quadruplicates.

	carbachol	iperoxo	NDMC	oxotremorine
**G_{i1}**	4.10±0.04	6.16±0.05	6.34±0.09	4.68±0.02
**G_{i2}**	4.06±0.00	6.14±0.03	6.26±0.01	4.67±0.03
**G_{i3}**	4.10±0.04	6.13±0.02	6.33±0.08	4.69±0.01
**G_{oA}**	4.07±0.01	6.15±0.04	6.24±0.01	4.71±0.01
**G_{oB}**	4.10±0.04	6.14±0.03	6.24±0.01	4.73±0.03

Table S3 Results of fitting Black & Leff equations to the experimental data

Experimental data were normalized to maximal system response (Figure 7). Black & Leff (Eq. 7) and Hill (Eq. 11) equations were fitted to the experimental data with E_{MAX} fixed to 1. *, different from Black & Leff ($p < 0.05$) according to ANOVA and Tukey HSD post-test.

	Black & Leff				Hill		
	**pK_A**	**τ**	**n**	**τ^n**	**pK_A**	**τ**	**n_H**
**G_{i1}**							
carbachol	4.38±0.03	1.55±0.04	1.00±0.03	1.55	4.59±0.01*	1.56±0.01	0.99±0.02
iperoxo	6.5±0.1	10±2	0.78±0.04	6.03	6.71±0.03	7.9±0.1	0.77±0.03
NDMC	6.5±0.1	0.26±0.07	1.5±0.3	0.132	7.22±0.02*	0.128±0.002*	1.25±0.07

	Black & Leff					Hill	
	pK_A	τ	n	$τ^n$	pK_A	τ	n_H
oxotremorine	5.17±0.04	2.5±0.1	0.83±0.02	2.14	5.46±0.02*	2.22±0.03*	0.88±0.02
**G_{i2}**							
carbachol	4.43±0.07	1.53±0.08	1.12±0.08	1.61	4.58±0.02	1.56±0.02	1.09±0.04
iperoxo	6.5±0.2	9±4	0.84±0.07	6.33	6.20±0.02*	17.9±0.2*	0.74±0.02
NDMC	6.21±0.07	0.21±0.03	1.19±0.12	0.156	6.96±0.02*	0.158±0.002*	1.07±0.05
oxotremorine	5.0±0.2	6.2±1.7	0.94±0.08	5.56	4.92±0.04	7.9±0.2	0.87±0.04
**G_{i3}**							
carbachol	4.47±0.03	1.12±0.02	0.92±0.03	1.11	4.81±0.01*	1.12±0.01	0.95±0.02
iperoxo	6.5±0.2	14±5	0.81±0.05	8.48	6.35±0.02	20.6±0.2*	0.75±0.02
NDMC	6.4±0.2	0.26±0.08	1.5±0.4	0.133	7.07±0.02*	0.131±0.002*	1.27±0.08
oxotremorine	5.6±0.1	0.25±0.03	0.69±0.06	0.384	6.33±0.04*	0.41±0.01*	0.78±0.04
**G_{oA}**							
carbachol	5.07±0.05	1.63±0.06	0.97±0.04	1.61	5.30±0.02*	1.62±0.02	0.98±0.03
iperoxo	7.1±0.1	3.9±0.6	0.51±0.02	2.00	7.59±0.03*	3.09±0.04*	0.53±0.01
NDMC	6.29±0.07	0.38±0.02	0.74±0.05	0.489	6.95±0.02*	0.509±0.006*	0.82±0.02
oxotremorine	5.57±0.08	2.8±0.3	0.55±0.02	1.76	6.17±0.03*	2.15±0.03*	0.63±0.01
**G_{oB}**							
carbachol	4.50±0.07	1.7±0.1	0.62±0.02	1.39	5.00±0.04*	1.68±0.03	0.68±0.02
iperoxo	7.1±0.1	3.9±0.7	0.53±0.03	2.06	7.70±0.05*	2.59±0.06*	0.61±0.03
NDMC	5.9±0.1	0.16±0.02	0.58±0.05	0.345	6.78±0.06*	0.38±0.01*	0.71±0.04
oxotremorine	5.5±0.2	1.8±0.4	0.52±0.05	1.36	5.65±0.08	2.67±0.01*	0.50±0.02

Python scripts

Scripts to model functional-response data and fit equations of Black & Leff, Hill and explicit models to them are provided in python.zip.

Requirements: python, numpy, matplotlib, scipy

Content of python.zip:

Folders:

- Black-Leff
- Hill
- OMA_low-expression_FR
- OMA_low-expression_Ke
- OMA_NCI

- OMA_Rtot

Black-Leff
Black-Leff_generate_5_data_sets.py generates 5 datasets named Data_A.dat through Data_E.dat according to the Black & Leff operational model of agonism (OMA) with parameters in the header of the script. Parameters of the model are saved in Black-Leff.par.

Hill
Hill_generate_5_data_sets.py generates 5 datasets named Data_A.dat through Data_E.dat according to the OMA modified with the Hill coefficient with parameters in the header of the script. Parameters of the model are saved in Hill.par.

OMA_low-expression_FR
OMA_low-expression_FR_generate_5_data_sets.py generates 5 datasets of functional response named Data_A.dat through Data_E.dat according to the OMA of a system with a similar expression of receptor and effector with parameters in the header of the script. Parameters of the model are saved in OMA_low-expression_FR.par.

Black-Leff_fit.py fits the Black & Leff OMA to the data. Fit results are saved in Black-Leff_fit.res Fit curves are saved as *_Black-Leff_fit.data.

Hill_fit.py fits the OMA modified with the Hill coefficient to the data. Fit results are saved in Hill_fit.res Fit curves are saved as *_Hill_fit.data.

OMA_low-expression_Ke
OMA_low-expression_Ke_generate_5_data_sets.py generates 5 datasets of signal transduction named Data_A.dat through Data_E.dat according to the OMA of a system with a similar expression of receptor and effector with parameters in the header of the script. Parameters of the model are saved in OMA_low-expression_Ke.par.

Black-Leff_fit.py fits the Black & Leff OMA to the data. Fit results are saved in Black-Leff_fit.res Fit curves are saved as *_Black-Leff_fit.data.

Hill_fit.py fits the OMA modified with the Hill coefficient to the data. Fit results are saved in Hill_fit.res Fit curves are saved as *_Hill_fit.data.

OMA_NCI
OMA_NCI_generate_5_data_sets.py generates 5 datasets named Data_A.dat through Data_E.dat according to the OMA of non-competitive auto-inhibition with parameters in the header of the script. Parameters of the model are saved in OMA_NCI.par.

Black-Leff_fit.py fits the Black & Leff OMA to the data. Fit results are saved in Black-Leff_fit.res Fit curves are saved as *_Black-Leff_fit.data.

Hill_fit.py fits the OMA modified with the Hill coefficient to the data. Fit results are saved in Hill_fit.res Fit curves are saved as *_Hill_fit.data.

OMA_NCI_fit.py fits the OMA of non-competitive auto-inhibition to the data. Fit results are saved in OMA_NCI_fit.res Fit curves are saved as *_OMA_NCI_fit.data.

OMA_NCI_Rtot

OMA_NCI_Rtot_generate_5_data_sets.py generates 5 datasets named Data_A.dat through Data_E.dat according to the OMA of non-competitive auto-inhibition with a varying number of receptors Rtot with parameters in the header of the script. Parameters of the model are saved in OMA_NCI.par.

Black-Leff_fit.py fits the Black & Leff OMA to the data. Fit results are saved in Black-Leff_fit.res Fit curves are saved as *_Black-Leff_fit.data.

Hill_fit.py fits the OMA modified with the Hill coefficient to the data. Fit results are saved in Hill_fit.res Fit curves are saved as *_Hill_fit.data.

OMA_NCI_Rtot_fit.py fits the OMA of non-competitive auto-inhibition with varying Rtot to the data. Fit results are saved in OMA_NCI_Rtot_fit.res Fit curves are saved as *_OMA_NCI_Rtot_fit.data.
